# FMRP Long-Range Transport and Degradation Are Mediated by Dynlrb1 in Sensory Neurons

**DOI:** 10.1016/j.mcpro.2023.100653

**Published:** 2023-09-21

**Authors:** Sara Emad El-Agamy, Laurent Guillaud, Keiko Kono, Yibo Wu, Marco Terenzio

**Affiliations:** 1 Molecular Neuroscience Unit, Okinawa Institute of Science and Technology Graduate University, Kunigami-gun, Okinawa, Japan; 2 Membranology Unit, Okinawa Institute of Science and Technology Graduate University, Kunigami-gun, Okinawa, Japan; 3 YCI Laboratory for Next-Generation Proteomics, RIKEN Center of Integrative Medical Sciences, Yokohama, Kanagawa, Japan; 4 Chemical Biology Mass Spectrometry Platform (ChemBioMS), Faculty of Science, University of Geneva, Geneva, Switzerland

**Keywords:** FMRP, RNA-binding protein, RNA granules, dynein, mRNA translation

## Abstract

The fragile X messenger ribonucleoprotein 1 (FMRP) is a multifunctional RNA-binding protein implicated in human neurodevelopmental and neurodegenerative disorders. FMRP mediates the localization and activity-dependent translation of its associated mRNAs through the formation of phase-separated condensates that are trafficked by microtubule-based motors in axons. Axonal transport and localized mRNA translation are critical processes for long-term neuronal survival and are closely linked to the pathogenesis of neurological diseases. FMRP dynein-mediated axonal trafficking is still largely unexplored but likely to constitute a key process underlying FMRP spatiotemporal translational regulation. Here, we show that dynein light chain roadblock 1 (Dynlrb1), a subunit of the dynein complex, is a critical regulator of FMRP function. In sensory axons, FMRP associates with endolysosomal organelles, likely through annexin A11, and is retrogradely trafficked by the dynein complex in a Dynlrb1-dependent manner. Moreover, Dynlrb1 silencing induced FMRP granule accumulation and repressed the translation of microtubule-associated protein 1b, one of its primary mRNA targets. Our findings suggest that Dynlrb1 regulates FMRP function through the control of its transport and targeted degradation.

## Highlights

• FMRP granules undergo axonal transport with lysosomes in mouse sensory neurons.
• Dynlrb1 mediates the association between FMRP and cytoplasmic dynein.
• Retrograde axonal transport regulates FMRP granule degradation.
• Dynlrb1 depletion induces FMRP granule accumulation and represses Map1b translation.

## Graphical Abstract

We performed a screening for dynein light chain roadblock 1 (Dynlrb1) interactors in mouse sensory neurons using proximity-based proteomics. We identified the fragile X messenger ribonucleoprotein 1 (FMRP), an RNA-binding protein implicated in neurological disorders, as a Dynlrb1’s interactor and showed that it undergoes retrograde axonal transport with lysosomes in sensory neurons. Dynlrb1 mediates FMRP function by controlling its transport and targeted degradation. Dynlrb1 genetic depletion causes the accumulation of FMRP granules, resulting in the repression of the translation of the microtubule-associated protein 1b, one of the primary mRNA targets of FMRP.

**Figure:**
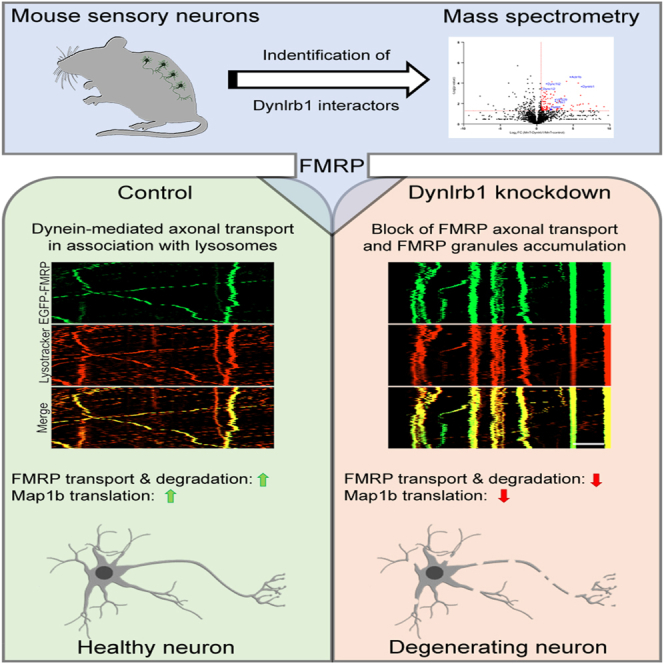

## In Brief

We identified the fragile X messenger ribonucleoprotein 1 (FMRP) as one of dynein light chain roadblock 1 (Dynlrb1) interactors *via* proteomic analysis. FMRP granules are transported in axons of sensory neurons together with lysosomes, and Dynlrb1 mediates the association between FMRP and cytoplasmic dynein. Long-range axonal transport is critical for the regulation of FMRP granule degradation, and its alteration *via* genetic depletion of Dynlrb1 induces FMRP granule accumulation and consequently represses the translation of the microtubule-associated protein 1b.

Cytoplasmic dynein 1 (hereafter referred to as dynein) is the main minus end–directed motor protein responsible for the retrograde transport of a variety of cargos, organelles, and cellular components in eukaryotic cells (1, 2), and its mutations have been linked to neurodegenerative diseases such as amyotrophic lateral sclerosis (ALS) (3, 4, 5, 6, 7, 8, 9). The dynein complex is built around the heavy chain, which, in addition to motility generation, provides a platform for the other subunits such as the intermediate, light–intermediate, and light chains that collectively mediate the direct and indirect association of cargos (2, 10). Dynein light chain roadblock 1 (Dynlrb1), the least studied subunit of the complex, was first identified in *Drosophila*, where its mutants displayed defects in intracellular transport, consequent severe axonal degeneration, and aberrant chromosomal segregation (11). Mammalian Dynlrb1 has been shown to work as an adaptor linking specific cargos to the dynein complex, including SMAD2 complexes activated by transforming growth factor beta receptors, Rab6 and *N*-acetyl-d-glucosamine kinase (12, 13, 14, 15, 16, 17, 18). Dynlrb1 was recently shown to be essential for neuronal survival, both during development and adulthood (19). Complete knockout of Dynlrb1 resulted in embryonic lethality, whereas conditional viral-mediated genetic reduction of Dynlrb1 in adult animals caused a loss of proprioceptive neurons, which could be at least partly explained by the observed impairment of endolysosomal trafficking (19).

Interestingly, one of the cargos transported by the dynein complex is mRNA, complexed with RNA-binding proteins (RBPs) into RNA granules (20, 21, 22). RBPs control the fate of their target mRNAs including their stability, translation, and localization (20, 23). Growing evidence suggests that altered translation of specific mRNAs, as a result of mutation or altered expression of RBPs, is a key pathogenic mechanism underlying a variety of neurological disorders (24, 25). One notable RBP, whose mutations are causative of fragile X syndrome (FXS), and fragile X-associated tremor/ataxia syndrome (FXTAS) (23, 26, 27), is the recently renamed fragile X messenger ribonucleoprotein 1 (FMRP) (28). FXS is a neurodevelopmental disease mainly characterized by intellectual disability, whereas FXTAS is a progressive neurodegenerative disease associated with tremors, cerebellar ataxia, and cognitive deficits. FMRP plays a crucial role in neuronal morphogenesis, cytoskeleton structure, synapse architecture, and synaptic transmission, mainly through mediating the localization and translation of its target mRNAs (23, 29, 30, 31, 32, 33, 34, 35). These functions are dependent on FMRP trafficking by molecular motors as well as its dynamic assembly and disassembly into neuronal granules through liquid–liquid phase separation (LLPS) (36, 37).

Given the critical role of Dynlrb1 in adult sensory neuron survival, we utilized a proximity labeling approach coupled with mass spectrometry (MS) to identify Dynlrb1 interactors. Proteomics analysis identified FMRP as one of the significant hits, and further analyses support a model in which Dynlrb1 silencing promotes FMRP granule formation, hence sequestering its target mRNAs and reducing their availability for translation. In addition, we show that FMRP granules cotraffic with LysoTracker-positive endolysosomal compartments (hereafter referred to as lysosomes for simplicity) and that Dynlrb1 is required to sustain their motility in cultured sensory neurons. Understanding the role of Dynlrb1 in the regulation of neuronal ribonucleoprotein (RNP) granule dynamics and transport opens a new perspective for understanding mRNA localization and translation and has implications for how Dynlrb1 could contribute to the development or progression of neurological diseases.

## Experimental Procedures

### Animal Experiments

All experiments involving animal subjects were carried out in accordance with the guidelines and regulations of Okinawa Institute of Science and Technology (OIST) Graduate University and approved by OIST Animal Care and Use Committee (protocol no.: 2020-304). Adult (8–10 weeks) male ICR mice were purchased from Jackson Laboratory. Animals were housed at 24.0 ± 0.5 °C with alternating 12 h day–night cycles and allowed access to food and water ad libitum.

### Reagents, Chemicals, and Antibodies

Culture media, sera, and chemicals were purchased from GibcoBRL, Invitrogen, and Sigma–Aldrich, respectively, unless specified otherwise. Drugs used in this study include puromycin (Sigma–Aldrich; catalog no.: P8833), anisomycin (Nacalai Tesque; catalog no.: 03046-14), leupeptin (Sigma–Aldrich; catalog no.: L2884), pepstatin A (Sigma–Aldrich; catalog no.: P5318), E-64d (Sigma–Aldrich; catalog no.: E8640), and MG132 (Sigma–Aldrich; catalog no.: M8699). Primary antibodies used in this study are anti-Tyr-α-tubulin (Synaptic Systems GmbH; catalog no.: 302117), anti-βIII-tubulin (Synaptic Systems GmbH; catalog no.: 302304), anti-FMRP (Invitrogen; catalog no.: PA534584 & OTI1C6 clone, catalog no.: TA504290), anti-dynein intermediate chain (Chemicon International, clone IC74.1; catalog no.: MAB1618), anti-flag (Sigma–Aldrich, clone M2; catalog no.: F3165), anti–vacuolar protein sorting–associated protein 29 (Vps29) (Abcam; catalog no.: ab236796), anti–vacuolar protein sorting–associated protein VTA1 homolog (Vta1) (Invitrogen; catalog no.: PA521831), anti-dynein heavy chain (Proteintech; catalog no.: 12345-1-AP), antipuromycin (Merck; catalog no.: MABE343), anti–microtubule-associated protein 1b (Map1b) (Invitrogen; catalog no.: PA582798), anti-β-actin (Sigma–Aldrich; catalog no.: A5441), anti-GFP (Roche; catalog no.: 11814460001), anti-G3BP stress granule assembly factor 1 (G3bp1) (Sigma–Aldrich; catalog no.: G6046), anti-lysosomal-associated membrane protein 1 (Lamp1) ([D2D11] XP, Cell Signaling Technology; catalog no.: 9091S), and anti–annexin A11 (Anxa11) (Proteintech; catalog no.: 68089-1-IG). Secondary antibodies used for immunostaining are anti-mouse Alexa Fluor 488 (Invitrogen; catalog no.: A11001), anti–guinea pig Alexa Fluor 568 (Invitrogen; catalog no.: A11075), anti-rat Alexa Fluor (Invitrogen; catalog no.: A-11077), anti-rabbit Alexa Fluor 568 (Invitrogen; catalog no.: A11011), anti–guinea pig Alexa Fluor 647 (Invitrogen; catalog no.: A21450), anti-rabbit Alexa Fluor 647 (Invitrogen; catalog no.: A21245), and streptavidin Alexa Fluor 647 conjugate (Invitrogen; catalog no.: S21374). Secondary horseradish peroxidase (HRP)–conjugated mouse and rabbit antibodies for immunoblots were anti-mouse HRP-conjugated antibody (Cell Signaling; catalog no.: 7076), goat anti-mouse (Invitrogen; catalog no.: G-21040), goat anti-rabbit (Abcam Limited; catalog no.: ab6721), and anti–streptavidin HRP (Cell Signaling Technology; catalog no.: 3999S).

### Dorsal Root Ganglia Neuronal Culture

Coverslips/dishes were coated with 0.01% poly-l-lysine (Sigma–Aldrich; catalog no.: P4832) overnight at 4 °C, rinsed with water, dried, and coated with laminin (GibcoBRL; catalog no.: 23017-015) for 1 h at 37 °C. Dorsal root ganglia (DRG) neuronal cultures were prepared as previously described (38). Briefly, DRG from all segmental levels were collected in Hanks’ balanced salt solution (HBSS) (GibcoBRL; catalog no.: 14175095), supplemented with 5 mM HEPES (Sigma–Aldrich; catalog no.: H0887), and 0.1 mg/ml primocin (Invivogen; catalog no.: ant-pm-1). Extracted DRG were enzymatically dissociated with 100 U of papain (Worthington Biochemical Corporation; catalog no.: PAP) in HBSS for 30 min at 37 °C followed by another 30 min of incubation in collagenase (1 mg/ml) and dispase (1.2 mg/ml) in HBSS at 37 °C (Worthington Biochemical Corporation; catalog no.: CLS2) (Roche Diagnostics GmbH; catalog no.: 04942078001). The ganglia were triturated in HBSS with 5 mM HEPES and 0.1 mg/ml primocin. Neurons were separated using a 20% percoll (Sigma–Aldrich; catalog no.: P4937) in L15 medium (GibcoBRL; catalog no.: L-5520), supplemented with 5 mM HEPES, 10% fetal bovine serum (FBS) (Invitrogen; catalog no.: 10270106), and 0.1 mg/ml primocin. Percoll gradient centrifugation was performed at 1000 rpm for 8 min. Neurons were plated in F-12 medium (Invitrogen; catalog no.: 11765054) supplemented with 10% FBS and 0.1 mg/ml primocin. After 2 days *in vitro* (DIV), the medium was supplemented with 5 μM of arabinofuranosylcytosine (Jena Bioscience GmbH; catalog no.: N-20307-1) to inhibit glial proliferation. Neurons were fixed for immunostaining or lysed for protein or RNA extractions at DIV 6 to 8 as described in the following sections unless specified otherwise.

### Human Embryonic Kidney Cells and Adeno-Associated Virus Production

Human embryonic kidney 293T cells (American Type Culture Collection) were maintained in Dulbecco's modified Eagle's medium (Nacalai Tesque; catalog no.: 08458-45) supplemented with 10% FBS. Adeno-associated virus (AAV) particles (PHP.S serotype (39)) were produced as previously described (40) with minor modifications. Briefly, human embryonic kidney cells were seeded to 70% confluency 24 h before transfection. A DNA mixture (gene of interest, ΔF6 helper, and PHP.S capsid plasmids) was introduced into the cells through a calcium phosphate transfection protocol (41). Four days later, cells were detached and suspended in artificial cerebrospinal fluid (124 mM NaCl, 3 mM KCl, 26 mM NaHCO_3_, 2 mM CaCl_2_.2H_2_O, 1 mM MgSO_4_.7H_2_O, 1.25 mM KH_2_PO_4_, and 10 mM d-glucose). AAV particles were retrieved through four freeze–thaw cycles, treated with Benzonase nuclease (Merck; catalog no.: 70664-3) at 45 °C for 15 min, and further purified from the crude lysate through multiple rounds of centrifugation at 14,000 rpm for 10 min at 4 °C. Purified AAV particles were used to infect primary neuronal cultures 3 h after plating.

### NIH/3T3 Cell Line Culture and Transfection

The mouse fibroblast cell line was acquired from Riken BRC Cell Bank (RCB2767) and cultured in a humidified incubator at 37 °C in low glucose Dulbecco's modified Eagle's medium (Fujifilm Wako; catalog no.: 041-29775) containing 10% FBS. Transient transfection was performed with Lipofectamine 3000 (Invitrogen; catalog no.: L3000015) according to the manufacturer’s instructions. Forty-eight hours after transfection, 3T3 cells were either fixed for 25 min in 4% paraformaldehyde (PFA) (Electron Microscopy Sciences; catalog no.: 15710) in PBS at room temperature (RT) for immunostaining or processed for protein extraction. Cells were lysed in 150 mM NaCl, 50 mM Tris (pH 7.4), 1% NP-40, 1 mM EDTA supplemented with complete protease inhibitor cocktail (Roche Diagnostics GmbH; catalog no.: 4693132001) for 30 min on ice. Lysates were clarified by centrifugation at 12,500 rpm for 15 min at 4 °C. The supernatant was collected and used for downstream experiments.

### Plasmid DNA

All recombinant DNA experiments were carried out in accordance with the guidelines and regulations of OIST genetic manipulation procedures and approved by the Biosafety Committee (protocol number: RDE-2020-013-4). Dynlrb1 gene was subcloned using an mRNA library isolated from the mouse brain using the RNeasy Mini kit (QIAGEN). The mRNA was then retrotranscribed in complementary DNA (Superscript III; ThermoFisher), and Dynlrb1 was amplified by PCR and inserted into a pcDNA3.1 vector *via* digestion with the restriction enzymes BamHI and XhoI. The primers used to amplify and clone Dynlrb1 in the pcDNA3.1 vector were the following:

Forward: CGGGATCCATGGCAGAGGTGGAGGAAAC

Reverse: CGCTCGAGTTCAGTTCAGTTGGATTCTGGATCAC

N- and C-terminal constructs with the Dynlrb1 sequence fused to flag tag and the miniTurbo (MnT) enzyme (42) in pcDNA3.1 were cloned by restriction-free cloning. A minimal linker (G4S1) between Dynlrb1 and MnT was used.

The primers used to amplify and clone Dynlrb1 in the MnT vector are the following:

**1. Dynlrb1 cloned at the C-terminus of MnT:**
Forward: CGATGACGACAAGCTTGCGGCCGCGATGGCAGAGGTGGAGGAAACACT
Reverse: CGGGATGCTGGATCCGCCTCCGCCTTCAGTTGGATTCTGGATCACAATCAGGA

**2. Dynlrb1 cloned at the N-terminus of MnT:**
Forward: GCCCAAAAGGGCGGAGGCGGATCCATGGCAGAGGTGGAGGAAACAC
Reverse: ATGCCACCCGGGATGATATCCCTCTAGAGTCGAGTTATTCAGTTGGATTCTGGATCACAATCAGG

The N-terminal fusion protein sequence was then subcloned using restriction digestion (SgsI and EcoRV) in an AAV vector backbone under the human synapsin I promoter (hSynI) (43, 44).

For shRNA-mediated knockdown experiments, “AAV-shRNA-ctrl” vector, a gift from Hongjun Song (Addgene, plasmid #85741 (45)), was used as a non-targeting control. To generate shDynlrb1 vector, the shControl sequence was deleted using BamHI and XbaI restriction enzymes, and a sequence targeting *Mus musculus* Dynlrb1 was inserted and ligated to the digested vector. The primers used to insert shDynlrb1 are as follows:

Forward: GATCCGATTATGGTGGCACCAGATAAGAAGCTTGTTATCTGGTGCCACCATAATCTTTTTTT

Reverse: CTAGAAAAAAAGATTATGGTGGCACCAGATAACAAGCTTCTTATCTGGTGCCACCATAATCG

shRNA constructs also express enhanced YFP (EYFP). All plasmids were verified by DNA sequencing (SeqStudio; Applied Biosystems) and were delivered to neurons using AAV particles. “p-EGFP-C1-flag-mFmr1(wt)” plasmid was a gift from Stephanie Ceman (Addgene, plasmid #87929 (46)) and was transfected using jetPEI (Polyplus; catalog no.: 101-10N) to express enhanced GFP (EGFP)–FMRP for axonal transport experiments.

### Axoplasm Pulldown

Axoplasm from mouse sciatic nerve was extracted as previously described (47). Briefly, sciatic nerves from two mice were dissected and mechanically squeezed out in isotonic buffer (20 mM HEPES, 110 mM KAc, 5 mM MgAc, 0.5 mM EGTA, pH 7.4). The axoplasm was then centrifuged at 4 °C for 15 min at 12,000 rpm to remove sciatic nerve fragments, and the supernatant was incubated overnight at 4 °C with 5 μg of anti-FMRP antibody (Invitrogen; catalog no.: PA534584) or rabbit immunoglobulin G isotype control (Cell Signaling; catalog no.: 2729) conjugated to 50 μl protein-G coupled magnetic beads (Dynabeads; ThermoFisher; catalog no.: 10003D) according to the manufacturer’s instructions. The beads were washed twice in isotonic buffer (20 mM HEPES, 110 mM KAc, 5 mM MgAc, 0.5 mM EGTA, pH 7.4) at 4 °C for 20 min and twice in PBS with 0.02% Tween-20 at 4 °C for 20 min. Proteins were eluted from the bead by boiling in 2× Laemmli sample buffer and loaded on 4 to 12% Bis–Tris plus gel (Invitrogen; catalog no.: NW04122BOX) and subsequently transferred to a nitrocellulose membrane (Invitrogen; catalog no.: LC2000) for Western blot analysis. The membrane was blocked with 5% bovine serum albumin (BSA) (Nacalai Tesque; catalog no.: 01860-65) for 30 min at RT and incubated with anti-FMRP (Invitrogen; catalog no.: PA534584) or anti-dynein intermediate chain (Chemicon International; catalog no.: MAB1618) antibodies. After three washes with Tris-buffered saline (TBS)–Tween-20 (0.05%), goat anti-mouse (Invitrogen; catalog no.: G-21040) or goat anti-rabbit (Abcam Limited; catalog no.: ab6721) HRP-conjugated antibodies were applied for 1 h at RT. The signal was developed using ECL prime (Cytiva; catalog no.: RPN2232), and images were acquired using iBright FL 1500 imaging system (Invitrogen). The intensity of the FMRP bands was quantified using ImageJ (NIH) software.

### Coimmunoprecipitation (3T3)

3T3 cells were lysed using an ionic detergent–free lysis buffer (150 mM NaCl, 50 mM Tris [pH 7.4], 1% NP-40, and 1 mM EDTA) supplemented with protease inhibitor cocktail. Anti-flag antibody (Sigma–Aldrich, clone M2, catalog no.: F3165) was conjugated to protein-G coupled magnetic beads through a 45 min incubation at 4 °C. Consecutively, the beads were washed with PBS–Tween-20 (0.02%) to remove any unbound antibody and incubated with the lysates overnight at 4 °C. The beads were washed with PBS–Tween-20, and the immobilized immune complexes were eluted by boiling the beads in 2× Laemmli buffer at 95 °C for 5 min. The elute was loaded onto 4 to 15% Tris–glycine gels (Bio-Rad; catalog no.: 4561084), and proteins were subsequently transferred onto a polyvinylidene difluoride membrane (Bio-Rad; catalog no.: 1704272). The membrane was blocked with 5% BSA for 30 min at RT and incubated with anti-dynein intermediate chain (Chemicon International, clone IC74.1; catalog no.: MAB1618) or anti-flag (Sigma–Aldrich, clone M2; catalog no.: F3165) antibodies. After TBS–Tween-20 (0.1%) washes, horse antimouse HRP-conjugated antibody (Cell Signaling; catalog no.: 7076) was applied for 1 h. The signal was developed using ECL prime (catalog no.: RPN2232), and images were acquired using ChemiDoc MP Imaging System (Bio-Rad).

### Expression of MnT Constructs, Biotinylation, and Streptavidin Pulldown

Cultured DRG neurons from adult mice were transduced with AAVs driving the expression of MnT (MnT-control) or MnT fused to Dynlrb1 (MnT–Dynlrb1). Six days later, transduced and non-transduced neurons were fed with 200 μM of biotin (Sigma–Aldrich; catalog no.: B4501) and incubated at 37 °C for 2 h to initiate labeling. Cells were washed thoroughly with PBS before lysis to remove any excess biotin. Neurons were lysed in a buffer composed of 150 mM NaCl, 50 mM Tris (pH 7.4), 1% NP-40, 0.5% SDS, and supplemented with a protease inhibitor cocktail. Purification of Dynlrb1 interactors was performed using streptavidin-conjugated beads (ThermoFisher; catalog no.: 88117). Five hundred μl of beads were washed three times in a buffer composed of 150 mM NaCl, 50 mM Tris (pH 7.4), and 0.1% Tween-20. The beads were resuspended in the same buffer containing a protease inhibitor cocktail, and then lysates were added and incubated overnight. Subsequently, the supernatant was removed, and the beads were washed three times with lysis buffer, twice with 2 M urea in PBS with 0.1% NP-40, followed by a final wash in 50 mM Tris (pH 7.4). All washes and binding steps were performed at 4 °C on a rotator mixer. To effectively elute the biotinylated proteins, the beads were heated at 65 °C in a reducing elution buffer (2% SDS, 150 mM NaCl, 150 mM Tris [pH 8.0], and 10 mM DTT) for 30 min.

### Experimental Design and Statistical Rationale for MS

The proteomics experiment was designed to identify Dynlrb1 interactors using a proximity-dependent biotinylation approach. Neurons transduced with a vector expressing only the MnT enzyme, and an untransduced negative control were adopted as experimental controls for proteins randomly biotinylated by the MnT enzyme, endogenously biotinylated proteins, and non-specific binding to the streptavidin beads, respectively. A trial MS run was performed first in the NIH/3T3 cell line and then in primary sensory neurons to calibrate the system. Three biological replicates (every replicate was formed by pooling cultured DRG from five mice to increase the protein yield) were utilized to ensure enough statistical power of detection beyond biological variance. Two technical repeats (two repeated LC–MS/MS measurements for each biological replicate) were acquired to improve the identified proteome coverage, thus ensure optimal collection of peptide information as reported before (48, 49), and to reduce the influence of noise on the statistical analysis.

### MS Sample Preparation and Data Acquisition

Gel-aided sample preparation was used to generate proteomics samples (50). Briefly, after eluting biotinylated proteins in the presence of 10 mM DTT (Wako; catalog no.: 047-08973), samples were cooled down and immediately incubated with 55 mM iodoacetamide (Wako; catalog no.: 095-02151). Proteins were then copolymerized with acrylamide through incubating with acrylamide/Bis-acrylamide solution (Supelco; catalog no.: 01709) at a final concentration of 20%, in the presence of *N*, *N*, *N*', *N*'-tetramethylethylenediamine and ammonium persulfate (Sigma–Aldrich; catalog nos.: T9281 and GE17-1311-01). The formed microgel was shredded by pulse centrifugation through a plastic grid to increase the surface area for the removal of detergents and chaotropic agents. Proteins were digested with trypsin/lys-C cocktail (Promega; catalog no.: V5073) at 37 °C overnight. Peptides were extracted using TFA and acetonitrile (Wako; catalog nos.: 206-10731 and 018-19853) and consecutively desalted using C18 stage tips (51). For LC–MS/MS, the tryptic peptides were measured with data-dependent acquisition using a Q Exactive Plus Hybrid Quadrupole-Orbitrap Mass Spectrometer and an EASY nLC 1200 Liquid Chromatography System, together with a Nanospray Flex Ion Source (ThermoFisher). The peptides prepared from eluted pull-down samples were dissolved in 13.0 μl of 0.1% formic acid, 3% acetonitrile, and 97% water, and 5.0 μl were injected twice for two separate measurements. The peptides prepared from input samples were dissolved in 20.0 μl of 0.1% formic acid, 3% acetonitrile, 97% water, and 2.0 μl were used to determine the peptide concentrations with a Pierce quantitative colorimetric peptide assay kit (ThermoFisher). An average of 0.6 μg of peptides was measured for each sample with two technical replicates. An Acclaim PepMap 100 75 μm × 2 cm, nanoViper 1200 bar precolumn (ThermoFisher) and a 3 μm C18 particle, 75 μm inner diameter, 12 cm filling length, analytical column (Nikkyo Technos Co, Ltd) were used to measure the samples. The 2 h data-dependent acquisition method and LC conditions used to measure the samples have been described previously (52). Full MS spectra were acquired with a scan range of 380 to 1500 *m/z* at a resolution of 70,000. The automatic gain control target was set to 3e^6^ with a maximum injection time of 100 ms. MS2 scans were recorded for the topN 20 precursors at a resolution of 17,500, automatic gain control of 1e^5^, and a maximum injection time of 60 ms. The default charge state for the MS2 was set to 2, and a normalized collision energy of 27% was set for higher-energy collisional dissociation fragmentation. The intensity threshold was set to 1.3e^4^, charge exclusion of unassigned, 1, 6 to 8, >8, and the dynamic exclusion was set to 20 s.

### MS Data Analysis, Database Search, Statistical Analysis, and Quantification

MaxQuant (Max Planck Institute of Biochemistry, version 1.6.3.3) was used for identification and label-free quantification. Protein identities were assigned by searching against a UniProt *Mus musculus* database (UP000000589.fasta, 55,470 proteins, April 2021). The individual peptide mass tolerance option in MaxQuant was utilized (calculated as reported (53)), whereas the initial maximum precursor mass tolerances were set to 20 ppm in the first search and 4.5 ppm in the main search, and the fragment mass tolerance was set to 20 ppm. Minimum Andromeda scores for accepting an MS/MS identification were set to 0 for unmodified peptides and 40 for modified peptides as recommended by the software manual. We required a minimum peptide length of seven amino acids and limited the search to a maximum peptide mass of 4600 Da. Trypsin was selected for digestion with a maximum of two missed cleavages. The match between runs was selected; matching time window of 10 min, match ion mobility window of 0.05, alignment time window of 20 min, and alignment ion mobility of 1. The false discovery rate was set to 0.05 for peptides and proteins. Database search parameters included cysteine carbamidomethylation as a fixed modification, methionine oxidation, and protein N-terminal acetylation as variable modifications. MaxQuant output data were further processed using GraphPad Prism (GraphPad Software, Inc) and Microsoft excel software. Protein intensities were normalized by the average of the total abundance of all proteins in the corresponding sample. Mean intensities were generated by averaging the intensities of the individual runs (three biological replicates and two technical repeats/biological replicate) per experimental sample. *p* Values for mean intensities of MnT–Dynlrb1 and MnT-control samples were calculated using multiple Student's *t* tests (54). Abundance ratios were generated for Dynlrb1-fused MnT compared with MnT-control and log_2_ transformed. Proteins with abundance ratios ≥2 and *p* values ≤0.05 were considered potential Dynlrb1 interactors. Functional enrichment analysis was performed using the software suite g:Profile (https://biit.cs.ut.ee/gprofiler/gost) (51).

### Cytotoxicity Assay

Cell viability was evaluated using cytopainter cell viability assay kit (Abcam; catalog no.: ab176744) as per the manufacturer’s instructions. Briefly, DRG neurons were transduced with AAV harboring shControl or shDynlrb1. Six days after transduction, the neurons were incubated with the dye for 45 min at 37 °C and 5% CO_2_. Cells were washed to remove excess dye, incubated in Tyrode’s solution, and transferred to a stage-top incubation chamber (P-set2000; Pecon, catalog no.: 133-800 261) on an LSM 900 confocal microscope and live imaged with a 20× objective (plan apochromat numerical aperture [NA] = 0.8). The number of dead and live cells was counted to estimate the degree of cytotoxicity imparted by Dynlrb1 depletion.

### Immunofluorescence

Cells were fixed in 4% PFA for 25 min. Coverslips were washed with PBS, and cells were permeabilized and blocked in 0.3% Triton X-100 and 5% normal goat serum (Invitrogen; catalog no.: 10000C) in PBS for 30 min. The following primary antibodies were incubated overnight at 4 °C: anti-flag (Sigma–Aldrich, clone M2; catalog no.: F3165), anti-Tyr-α-tubulin (Synaptic Systems GmbH; catalog no.: 302117), anti-βIII-tubulin (Synaptic Systems GmbH; catalog no.: 302304), and anti-G3bp1 (Sigma–Aldrich; catalog no.: G6046). After washing, fluorescent secondary bodies were applied for 1 h at RT: anti-mouse Alexa Fluor 488 (Invitrogen; catalog no.: A11001), anti–guinea pig Alexa Fluor 568 (Invitrogen; catalog no.: A11075), streptavidin Alexa Fluor 647 conjugate (Invitrogen; catalog no.: S21374), anti-rat Alexa Fluor 568 (Invitrogen; catalog no.: A-11077), anti-mouse Alexa Fluor 647 (Invitrogen; catalog no.: A21236), and anti-rabbit Alexa Fluor 647 (Invitrogen; catalog no.: A21245). For visualization of somal and axonal FMRP granules, cells were fixed in 4% PFA overnight or for 25 min, respectively. Permeabilization was performed using 1% saponin in PBS for somatic granules and 0.1% saponin for axonal granules. Saponin solutions were freshly prepared and supplemented with 75 mM glycine as a quenching agent. Blocking was performed using 5% normal goat serum for 30 min. Saponin (0.1% and 0.01%) were maintained during blocking, washes, and antibody incubation steps for somal and axonal granules, respectively. The following antibodies were used: anti-FMRP (Invitrogen; catalog no.: PA534584) and anti-βIII-tubulin (Synaptic Systems GmbH; catalog no.: 302304), anti-rabbit Alexa Fluor 568 (Invitrogen; catalog no.: A11011), and anti–guinea pig Alexa Fluor 647 (Invitrogen; catalog no.: A21450). Coverslips were mounted with Fluoromount G or ibidi mounting media and imaged using Zeiss LSM 780 or 900 confocal microscopes using a 63× oil-immersion objective (plan apochromat NA = 1.4). For visualization, maximum intensity projection images were adjusted for brightness and contrast levels using ImageJ or Zen (Zeiss) software.

### Airyscan Image Acquisition and Analysis

Super-resolution images were acquired on LSM 900 with Airyscan 2.0 super-resolution module using a 63× oil-immersion objective (plan apochromat NA = 1.4). Initial image acquisition parameters were as follows: image size 1834 × 1834 pixels (78 × 78 μm), pixel resolution 0.043 × 0.043 × 0.150 μm/pixel, pixel dwell time 1.15 μs, laser power 1% (detector gain 900 V), and 0.4% (detector gain 850%) for laser line ex647 and ex568, respectively, and optimal z-section of 150 nm and around 20 to 30 stacks. Airyscan images were processed in Imaris 10 (Bitplane; Oxford Instruments). FMRP granules were identified using Imaris spot tracking plugin with an initial spot size detection of 150 nm and automatic background subtraction, and the spot region growth was based on their absolute intensity. Axons were identified using the surface rendering plugin for the tubulin channel with smooth filter and a surface grain size of 0.085 μm. FMRP granule size, number, and clustering were calculated using Imaris measurement pro plugin. FMRP area was estimated from spot volume, and clustering was estimated by measuring the average distance between three or nine neighboring FMRP spots per image.

### Proximity Ligation Assay

Proximity ligation assay (PLA) was performed using Duolink PLA reagents (Sigma–Aldrich) according to the manufacturer’s protocol. Cells were fixed, permeabilized, and blocked as described in the immunofluorescence section. The antibodies used for the semiendogenous PLA experiments were anti-flag (Sigma–Aldrich, clone M2; catalog no.: F3165), anti-FMRP (Invitrogen; catalog no.: PA534584), anti-Vps29 (Abcam; catalog no.: ab236796), and anti-Vta1 (Invitrogen; catalog no.: PA521831). For the endogenous PLA, antidynein heavy chain (Proteintech; catalog no.: 12345-1-AP), anti-FMRP (Invitrogen, OTI1C6 clone; catalog no.: TA504290), and anti-Lamp1 ([D2D11] XP, Cell Signaling Technology; catalog no.: 9091S) antibodies were used. The probes used were the anti-mouse minus and anti-rabbit plus probes (DUO92004 and DUO92002), and the signal was detected using the far-red detection kit (DUO92013). When indicated, cells were counterstained with anti-βIII-tubulin (Synaptic Systems GmbH; catalog no.: 302304) for 1 h at RT. Coverslips were mounted with Duolink mounting medium (DUO82040) and sealed with nail polish. Images were acquired using Zeiss LSM 900 confocal microscope using a 63× oil-immersion objective (plan apochromat NA = 1.4). PLA signal was quantified using ImageJ. The signal intensity was manually thresholded with the same value for controls and samples. The cell body size was manually outlined by the user, and the axonal network was defined by the βIII-tubulin or EYFP mask area. The number of puncta was calculated and divided by the cell body or the neuronal network area in maximum intensity projection images, after subtracting any areas of glia or cellular debris.

### Puromycinylation and Puro-PLA

Detection of newly synthesized Map1b was performed by incubating neuronal cultures with 5 μM puromycin (Sigma–Aldrich; catalog no.: P8833) in F-12 for 10 min at 37 °C in a 5% CO_2_ incubator. Protein synthesis inhibition control groups were preincubated with 40 μM of anisomycin (Nacalai Tesque; catalog no.: 03046–14) in F-12 for 30 min before the addition of puromycin. The incubation was terminated by two quick washes in PBS, and cells were fixed immediately using 4% PFA for 25 min. After permeabilization and blocking, PLA was performed as described using anti-puromycin (Merck; catalog no.: MABE343) and anti-Map1b (Invitrogen; catalog no.: PA582798) antibodies. Puro-PLA signal was quantified as previously described in the PLA section.

### RNAscope Multiplex Fluorescent Assay Combined With Immunofluorescence

The integrated co-detection workflow recommended by the manufacturer was followed with minor modifications. DRG neurons cultured on coverslips were fixed using fresh 4% PFA at RT for 30 min. After PBS washes, the samples were dehydrated through 1 min sequential incubations in 50%, 70%, and 100% ethanol and stored at −20 °C in 100% ethanol until the assay was performed. The cells were rehydrated through 1 min sequential incubations in 70%, 50% ethanol, and water. Samples were then permeabilized using PBS–Tween (0.1%) for 10 min, treated with hydrogen peroxide for 10 min, washed with water, and incubated with the primary antibodies (anti-FMRP [Invitrogen; catalog no.: PA534584] and anti-GFP [Roche; catalog no.: 11814460001]) overnight at 4 °C. The anti-GFP antibody was used to enhance the AAV-EYFP signal impacted by the downstream protease treatment. Cells were then washed three times in PBS–Tween (0.1%), postfixed using 4% PFA for 30 min, washed with PBS–Tween (0.1%), and treated with protease III (1:100 dilution, 10 min, RT). As reported by other groups (55), higher protease concentrations resulted in a significant loss of the primary antibody signal. Different protease concentrations did not have any influence on the mRNA signal obtained from primary cultures on coverslips (55). After five washes in PBS, the multiplex v2 fluorescent *in situ* hybridization (ISH) assay was run as per the manufacturer’s instructions. Briefly, the Map1b probe (Advanced Cell Diagnostics; catalog no.: 1045181-C3) or the negative (Advanced Cell Diagnostics; catalog no.: 320871) and positive control probes (Advanced Cell Diagnostics; catalog no.: 320881) were hybridized for 2 h at 40 °C. Then Amp1, Amp2, and Amp3 hybridization cycles were performed, followed by developing the C3-HRP channel with TSA vivid fluorophore 650 (Tocris; catalog no.: 323273). After the final HRP blocking step, secondary antibodies: anti-mouse Alexa Fluor 488 (Invitrogen; catalog no.: A11001) and anti-rabbit Alexa Fluor 568 (Invitrogen; catalog no.: A11011) were incubated for 30 min at RT. Coverslips were then washed with PBS–Tween (0.1%), PBS, mounted with Fluoromount G, and imaged using Zeiss LSM 900 confocal microscope using a 63× oil-immersion objective (plan apochromat NA = 1.4). The fractional area of overlap between FMRP and Map1b signal was quantified using ImageJ. Axonal FMRP and Map1b signals were thresholded manually with a matched value for all experimental groups, and the overlap area between the two channels was divided by the total FMRP area.

### Pharmacological Treatment

DRG neuron cultures, transduced with either shControl or shDynlrb1, were treated on DIV8 with lysosomal inhibitors (200 μM leupeptin, 20 μM pepstatin A, and 2 μM E-64d), a specific proteasomal inhibitor (MG132, 10 μM) or an equivalent volume of vehicles (dimethyl sulfoxide and water) for 6 h. Neurons were washed with PBS and lysed for detecting FMRP levels by Western blotting.

### Protein Extraction and Western Blotting

Neurons were lysed in radioimmunoprecipitation assay buffer (150 mM NaCl, 50 mM Tris [pH 8.0], 1% Triton X-100, 0.1% SDS, and 0.5% sodium deoxycholate) supplemented with protease inhibitor cocktail. Lysates were boiled with Laemmli buffer at 95 °C for 5 min, loaded onto 4 to 12% Bis–Tris gels (Invitrogen; catalog no.: NP0323BOX), and subsequently transferred to nitrocellulose or polyvinylidene difluoride membranes (Invitrogen; catalog nos.: LC2000 and LC2002). The blotted membranes were blocked in 5% BSA for 40 min at RT and then incubated with primary antibodies overnight at 4 °C. The following primary antibodies were used: anti-FMRP (Invitrogen; catalog no.: PA534584), anti-β-actin (Sigma–Aldrich; catalog no.: A5441), anti-flag (Sigma–Aldrich, clone M2; catalog no.: F3165), anti-GFP (Roche; catalog no.: 11814460001), and anti-Anxa11 (Proteintech; catalog no.: 68089-1-IG). Afterward, membranes were washed four times with TBS–Tween-20 (0.1%), and secondary HRP-conjugated antibodies were applied for 1 h at RT: goat anti-mouse (Invitrogen; catalog no.: G-21040), goat anti-rabbit (Abcam Limited; catalog no.: ab6721), and streptavidin HRP (Cell Signaling Technology; catalog no.: 3999S). After another set of 30 min TBS–Tween-20 washes, the chemiluminescence signal was developed using Cytiva ECL start detection reagents (RPN3243) and detected using iBright FL 1500 imaging system. The band’s intensity was quantified by ImageJ software.

### Gene Expression Analysis by Quantitative Reverse Transcription PCR

Total RNA was extracted and purified using NucleoSpin RNA plus kit (Macherey–Nagel; catalog no.: 740984.50). RNA concentration was assessed, and complementary DNA was synthesized according to the manufacturer’s instructions (QIAGEN; catalog no.: 205311). PCR amplification was performed using PowerUp SYBR Green Master Mix (Thermo Fisher Scientific; catalog no.: A25776), and target gene expression was measured using qTower^3^ real-time PCR system (Analytikjena). The following thermocycling parameters were used, an initial 20 s denaturation step followed by 40 two-step cycles, a denaturation step at 95 °C for 1 s, and a combined annealing–extension step at 60 °C for 30 s. Relative gene expression presented as fold change was calculated using the ΔΔCt method, where 18S ribosomal RNA was used as a reference gene to normalize the expression. The primers (*Mus musculus*) used were as follows (19):

18S forward: AAACGGCTACCACATCCAAG

18S reverse: CCTCCAATGGATCCTCGTTA

Dynlrb1 forward: CAACCTCATGCACAACTTCATC

Dynlrb1 reverse: TCTGGATCACAATCAGGAAATAGTC

### siRNA Transfection

Four hours after plating, DRG neurons were transfected with an siRNA pool targeting Anxa11 (Sigma–Aldrich; catalog no.: EMU006471), Dynlrb1 (Sigma–Aldrich; catalog no.: EMU089881), or a universal non-targeting siControl (Sigma–Aldrich; catalog no.: SIC001) using Dharmafect4 (Horizon Discovery; catalog no.: T-2004-03) according to the manufacturer’s protocol.

### Axonal Transport Experiments

To analyze the percentage of motile FMRP particles and LysoTracker carriers as well as their respective speeds, DRG neurons (wildtype, siControl, and siDynlrb1) were transfected with EGFP-FMRP (Addgene plasmid #87929 (46)) using JetPEI (Polyplus; catalog no.: 101-10N) according to the manufacturer’s instructions. On DIV3, 150 nM of LysoTracker Red DND-99 (Invitrogen; catalog no.: L7528) was added to the cells for 30 min at 37 °C and 5% CO_2_. The LysoTracker containing medium was washed out to remove the excess dye and replaced with Tyrode’s solution (Sigma–Aldrich; catalog no.: T2397). Neurons were transferred to a stage-top incubation chamber (P-set2000; Pecon; catalog no.: 133-800 261) on an LSM 900 confocal microscope and imaged with a 63× oil-immersion objective (plan apochromat NA = 1.4). Hundred and twenty frames (frame duration of 2.53 s) were consecutively acquired for every time series. To analyze mitochondrial dynamics, DRG neurons transfected with siControl or siDynlrb1 were incubated with 100 nM of MitoTracker Deep Red FM (Invitrogen; catalog no.: M22426) for 20 min at 37 °C and 5% CO_2_. Cultures were washed, incubated in Tyrode’s solution, and transferred to a stage-top incubation chamber (P-set2000, Pecon; catalog no.: 133-800 261) on an LSM 900 confocal microscope and imaged with a 63× oil-immersion objective (plan apochromat NA = 1.4). Hundred and ninety five frames (frame time of 1.27 s) were consecutively acquired for every time series. The percentage of stationary *versus* moving carriers was manually calculated on kymographs generated using Zen blue software (version 3.2). Statistical analysis was performed with two-way ANOVA using the GraphPad Prism software.

LysoTracker and FMRP transport movies were tracked manually using the Manual Tracking plugin of the Fiji software (https://imagej.net/software/fiji/). MitoTracker movies were tracked using a MATLAB script designed in house (56) and archived here at https://groups.oist.jp/mnu/productalgorithmsoftware.

### Statistical Analysis

All experiments were performed in at least three biological replicates. Analysis of multiple groups was performed using ANOVA. The choice between one- or two-way ANOVA was based on the requirements for identification of specific factors’ contribution to statistical differences between groups and were followed by Tukey’s and Sidak’s post hoc analysis tests, respectively. For two-group analyses, unpaired Student’s *t* test was used. All statistical analyses were performed using GraphPad Prism. Data are presented as mean ± SEM. Statistical significance tests and the number of samples used are described in the figure legends. Significance values are indicated as ∗*p* <0.05, ∗∗*p* <0.01, and ∗∗∗*p* <0.001; n.s. indicates not significant.

## Results

### Design and Optimization of Dynlrb1–MnT Fusion Constructs for Proximity Labeling

To identify Dynlrb1 interactome, we utilized a proximity-dependent biotinylation approach coupled with MS. We fused the MnT enzyme and a flag tag to both the N- and C- terminus of Dynlrb1. A vector expressing only the MnT enzyme was adopted as an internal control to allow for the exclusion of proteins that are randomly biotinylated in the cytoplasm, rather than being potential Dynlrb1 interactors. To reduce the number of animals in the study, we optimized the procedures for promiscuous protein biotinylation, purification, and identification in the 3T3 mouse cell line. To exclude the possibility that the fusion of the MnT could affect the folding of Dynlrb1 or its integration into the dynein complex, flag immunofluorescence staining, and coimmunoprecipitation assays were performed in 3T3 cells. Expression of the C-terminus fusion protein (Dynlrb1–MnT) in 3T3 cells resulted in its aggregation and reduced cell viability (supplemental Fig. S1*A*). In addition, while the flag immunoprecipitation efficiency was almost equivalent for both fusion proteins, dynein intermediate chain coprecipitated solely with the N-terminus protein (MnT–Dynlrb1) (supplemental Fig. S1*B*). Thus, we decided to use only the N-terminal fusion construct moving forward. We then tested the efficiency of biotinylation and pulldown in 3T3 cells transfected with MnT-control or MnT–Dynlrb1 constructs and treated with 200 μM of biotin for 2 h. Following streptavidin-mediated pulldown, we could retrieve most biotinylated proteins (supplemental Fig. S1*C*).

**Fig. 1:**
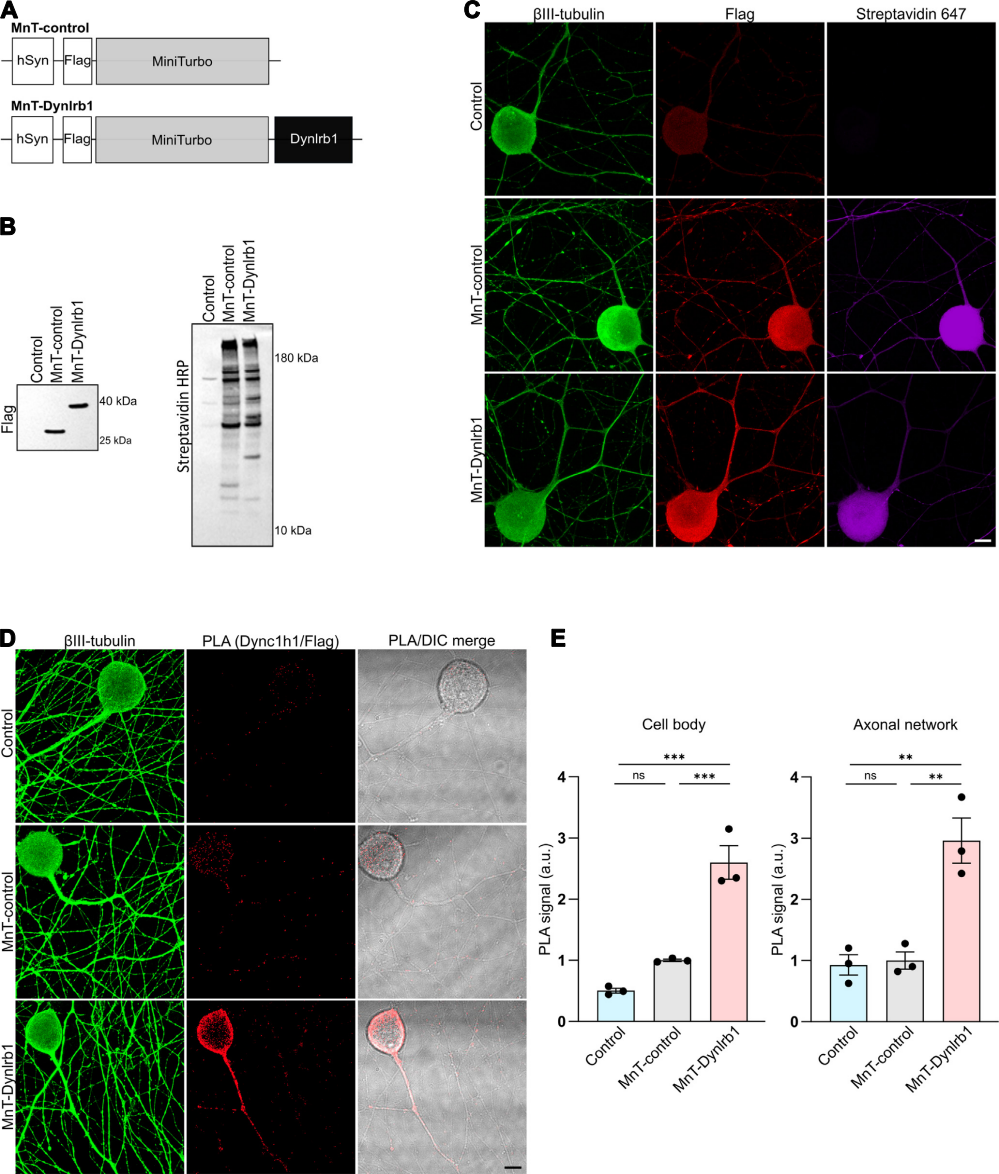
**Optimization of proximity labeling in cultured DRG neurons.***A*, schematic representation of the miniTurbo (MnT) constructs used to infect DRG neurons in this study. *B*, Western blot analysis of the expression and biotinylation pattern of MnT-control and MnT–Dynlrb1 detected using anti-flag and anti–streptavidin HRP antibodies, respectively. A non-transduced sample is included as a negative control. *C*, DRG neurons were transduced with MnT-control or MnT–Dynlrb1 and subjected to biotinylation. Non-transduced cells were also added as a control. Neurons are labeled with βIII-tubulin (*green*). The expression is revealed using an anti-flag antibody (*red*), whereas the extent of biotinylation is detected using streptavidin Alexa 647 (*magenta*). Scale bar represents 10 μm. *D*, PLA analysis between dynein heavy chain (Dync1h1) and flag-tagged proteins (MnT–Dynlrb1 and MnT-control). Non-transduced cells were included as a control. Neurons are labeled with βIII-tubulin (*green*). The PLA signal is in *red*. Scale bar represents 10 μm. *E*, quantification of the PLA experiment in (*D*). PLA signal in the cell bodies and axons was quantified separately. Mean ± SEM, ∗∗*p* < 0.01, ∗∗∗*p* < 0.001, ns, not significant, n = 3, one-way ANOVA followed by Tukey’s HSD post hoc correction for multiple comparisons. DRG, dorsal root ganglia; HRP, horseradish peroxidase; HSD, honestly significant difference test; PLA, proximity ligation assay.

We then transferred the MnT-control and the MnT–Dynlrb1 sequences to AAV vector backbone (Fig. 1*A*) to transduce cultured DRG neurons. Western blot analysis confirmed efficient expression of the flag-tagged proteins 6 days after infection (Fig. 1*B*). The activity of the enzyme in transduced cultures was confirmed by revealing the extent of biotinylation *via* Western blot analysis with anti–streptavidin HRP (Fig. 1*B*). We further validated the construct expression and ability to biotinylate proteins *via* immunofluorescence staining of transduced sensory neurons (Fig. 1*C*).

Furthermore, we took advantage of *in situ* proximity ligation assay (PLA), a technique that allows direct visualization of protein–protein close association (40 nm range (57)) with high specificity and sensitivity (58) to validate the interaction between MnT–Dynlrb1 and the dynein complex in neuronal cultures. Indeed, robust association between MnT–Dynlrb1 and dynein heavy chain (Dync1h1) was detected in both neuronal cell bodies and axons (Fig. 1, *D* and *E*). Thus, we confirmed MnT–Dynlrb1 incorporation in the dynein complex in DRG neurons.

### Identifying Dynlrb1 Interactors in DRG Neurons *via* MS

**Fig. 2:**
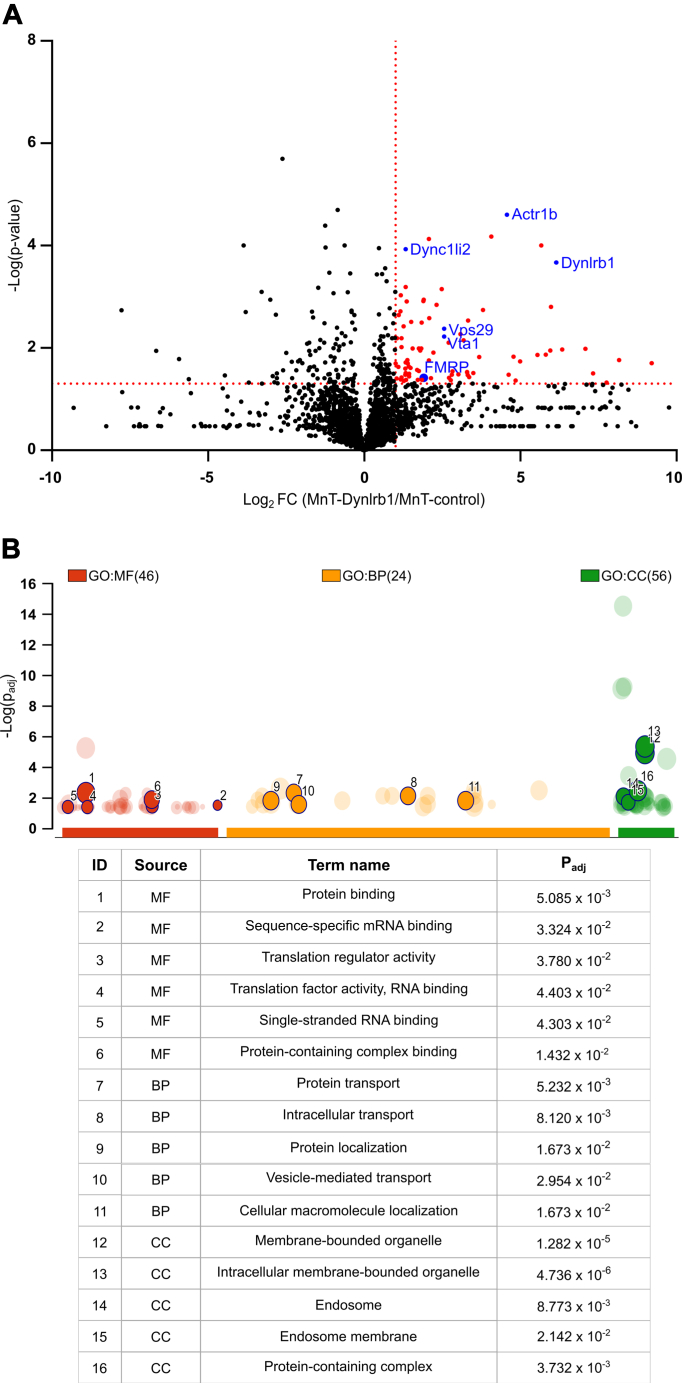
**Mass spectrometry (MS) analysis of Dynlrb1 interactors.***A*, Volcano plot of the MS analysis of DRG neurons transduced with MnT-control or MnT–Dynlrb1 AAV constructs. *Red* and *blue circles* represent putative interactors. *Vertical and horizontal dashed red lines* mark two-fold change (log_2_(2) = 1) compared with MnT-control and *p* value of 0.05 (−log_10_(0.05) = 1.3), respectively. *B*, Manhattan plot of the candidate hits from the MS analysis. Functional enrichment analysis was performed using the g:Profile server. GO categories associated with the MS hits are plotted *versus* the −log_10_ of the adjusted *p* values (Padj). *Circles* represent functional terms that are grouped and color coded by data source: BP, biological process; CC, cellular component; GO, gene ontology; MF, molecular function. A few chosen categories are highlighted in the Manhattan plot and table below the chart. AAV, adeno-associated virus; DRG, dorsal root ganglia; MnT, miniTurbo.

To identify Dynlrb1 interactors, cultured DRG neurons transduced with either MnT-control or MnT–Dynlrb1 constructs were treated with 200 μM biotin for 2 h to initiate labeling. Biotinylated proteins were captured by streptavidin magnetic beads, eluted, and subjected to LC–MS/MS. Subsequent MaxQuant analysis identified 90 Dynlrb1 interactors significantly enriched over MnT-control (fold change ≥ 2; *p* ≤ 0.05), including subunits of the dynein and dynactin complex (Fig. 2*A* and supplemental Tables S1 and S2). Bioinformatic analysis using the g:Profile server (https://biit.cs.ut.ee/gprofiler, (59)) highlighted an enrichment of proteins involved in protein transport as expected (Fig. 2*B*), confirming the successful pulldown of Dynlrb1-related dynein complexes. mRNA translation and RNA binding were also represented in the proteomics hits (Fig. 2*B*).

**Fig. 3:**
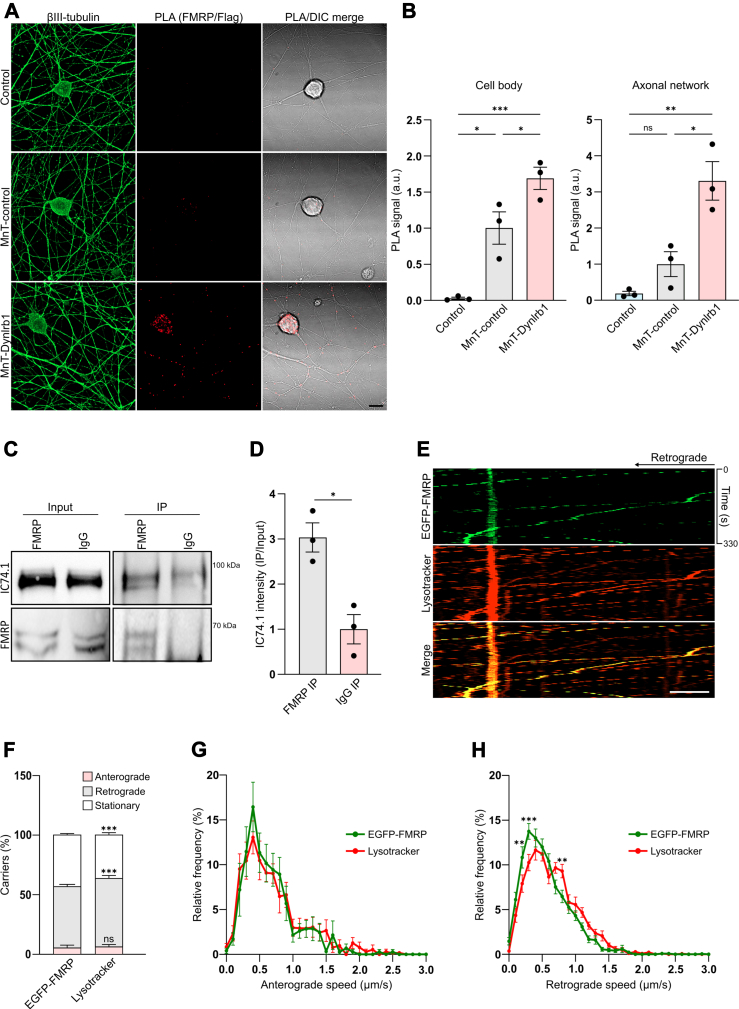
**FMRP interacts with dynein and is transported in axons together with lysosomes.***A*, representative images of PLA between FMRP and flag-tagged proteins in DRG neurons transduced with MnT-control or MnT–Dynlrb1. Non-transduced cells were also added as a control. Neurons are labeled with βIII-tubulin (*green*). The PLA signal is in *red*. Scale bar represents 10 μm. *B*, quantification of the PLA experiment in (*A*). PLA signal in the cell bodies and axons was quantified separately. Mean ± SEM, ∗*p* < 0.05, ∗∗*p* < 0.01, ∗∗∗*p* < 0.001, ns, not significant, n = 3, one-way ANOVA followed by Tukey’s HSD post hoc correction for multiple comparisons. *C*, Western blot analysis of FMRP immunoprecipitation from axoplasm extracted from mouse sciatic nerve. Coimmunoprecipitation of dynein intermediate chain is revealed by immunostaining with IC74.1 antibody. *D*, quantification of the pulldown in (*C*). Mean ± SEM, ∗*p* < 0.05, n = 3, unpaired *t* test. *E*, representative kymograph of DRG neurons transfected with EGFP-FMRP construct (in *green*). LysoTracker was added to track lysosomal transport (in *red*). The merge between the two channels is in *yellow*. Scale bar represents 5 μm. *F*, percentage of anterograde and retrograde *versus* stationary carriers in the experiment described in (*E*). *G*, anterograde speed distributions from the experiment described in (*E*). Mean ± SEM, n >14 movies per group over three independent biological repeats, two-way ANOVA, followed by Sidak's multiple comparisons test. *H*, retrograde speed distributions from the experiment described in (*E*). Mean ± SEM, ∗∗*p* < 0.01, ∗∗∗*p* < 0.001, n >28 movies per group over three independent biological repeats, two-way ANOVA, followed by Sidak’s multiple comparisons test. DRG, dorsal root ganglia; EGFP, enhanced GFP; FMRP, fragile X messenger ribonucleoprotein 1; HSD, honestly significant difference test; MnT, miniTurbo; PLA, proximity ligation assay.

We selected three candidates for validation based on their role in intracellular trafficking and neuronal homeostasis: the vacuolar protein sorting–associated protein 29 (Vps29), the vacuolar protein sorting–associated protein VTA1 homolog (Vta1), and FMRP. We used PLA to visualize the association between Dynlrb1 and the selected candidates as well as the subcellular localization of said interaction. Because of the lack of reliable antibodies against Dynlrb1, we performed our PLA analysis between the endogenous candidates and the flag-tagged MnT-control or MnT–Dynlrb1 proteins. Semiendogenous PLA signal was previously shown to represent a valid protein interaction (60). Reassuringly, all the candidate hits showed interaction with Dynlrb1 when compared with the MnT-control (Fig 3, *A* and *B* and supplemental Fig. S2). While Vps29–Dynlrb1 interaction was mainly axonal (supplemental Fig. S2, *A* and *B*), Vta1 and FMRP showed significant association with Dynlrb1 across both the somatic and axonal compartments (supplemental Fig. S2, *C* and *D* and Fig. 3, *A* and *B*). Because of the relevance of FMRP to neuronal pathology and the relative lack of data regarding its interaction with the dynein complex, we selected this candidate for further investigations.

### FMRP Associates With Dynein and Lysosomes in Axons of Sensory Neurons

Interestingly, FMRP has been reported to interact with bicaudal-D (BicD), a dynein-activating adaptor, driving its molecular transport into *Drosophila*’s dendrites (36). In addition, recent work demonstrated that RBPs complexed within RNA granules can gain indirect access to long-range transport through associating with already-motile Lamp1-positive endolysosomes, a process known as hitchhiking (61, 62). Our previous data indicates that Dynlrb1 is crucial for lysosomal transport in DRG neurons (19), thus we decided to characterize FMRP association with dynein and its axonal trafficking in relation to lysosomes in sensory axons.

To obtain enough axonal lysate for a coimmunoprecipitation assay, we isolated axoplasm from mouse sciatic nerve and immunoprecipitated FMRP. Indeed, we could recover dynein intermediate chain (IC74.1) in the FMRP pulldown (Fig. 3, *C* and *D*), confirming the association of FMRP with the dynein complex in axonal cytoplasm and suggesting the possibility of dynein-based axonal transport of FMRP. We then expressed EGFP-FMRP in cultured DRG neurons and performed time-lapse imaging together with LysoTracker DND-99, a dye that allows for live labeling of lysosomes. Our time-lapse imaging revealed that 56% and 63% of FMRP and endolysosomes, respectively, exhibited a processive directional movements in wildtype neurons (Fig. 3, *E* and *F*), in agreement with previous observations (19, 61). Interestingly, while most anterograde and retrograde motile EGFP-FMRP granules trafficked with lysosomes, not all lysosomes cotrafficked with FMRP granules (Fig. 3*E* and Movie S1). EGFP-FMRP positive carries exhibited a preferential retrograde trafficking in sensory axons (Fig. 3, *E* and *F*). Frequency distribution of EGFP-FMRP speed showed a characteristic peak in each direction (Fig 3, *G* and *H* and supplemental Fig. S3, *A* and *B*) with the anterograde peak closely aligning with that of LysoTracker-positive organelles (Fig. 3*G* and supplemental Fig. S3*A*), whereas the retrograde peak revealed a selective association with a subpopulation of endolysosomes (Fig. 3*H* and supplemental Fig. S3*B*). The observed retrograde bias in motility could support a model in which EGFP-FMRP is coupled with a subpopulation of LysoTracker-positive organelles for degradation in the soma. While local axonal degradation has been reported, the majority of enzymatically active degradative lysosomes reside in the soma (63, 64). Retrograde shuttling, as opposed to the bidirectional transport described for EGFP-FMRP in dendrites of other neuronal types, could be more prominent in DRG, whose network is exclusively axonal. Indeed, unlike dendrites, axons are characterized by a uniform plus-ended microtubule polarity, and dynein is a retrograde motor in this context (65, 66, 67).

Recently, Lippincott-Schwartz and Ward groups identified annexin A11 (Anxa11), a vesicular trafficking protein linked to ALS (68), as the tethering adaptor for G3BP stress granule assembly factor 1 (G3bp1) granule association with motile lysosomal-associated membrane protein 1 (Lamp1)-positive vesicles (62). Interestingly, Anxa11 was one of the hits identified in our proteomics screen (supplemental Tables S1 and S2). Thus, we examined the impact of Anxa11 silencing, using an siRNA pool, on the association of endogenous FMRP to Lamp1-positive organelles using PLA. The efficiency of the knockdown was confirmed by Western blot analysis (supplemental Fig. S3, *C* and *D*). Remarkably, PLA analysis revealed reduced FMRP association with Lamp1 organelles upon Anxa11 depletion across the somatic and axonal compartments (supplemental Fig. S3, *E*–*J*) suggesting that Anxa11 could mediate the docking of at least a pool of FMRP granules onto Lamp1-positive vesicles for long-range transport.

### FMRP Axonal Trafficking Correlates With Lysosomes and Depends on Dynlrb1 Levels

**Fig. 4:**
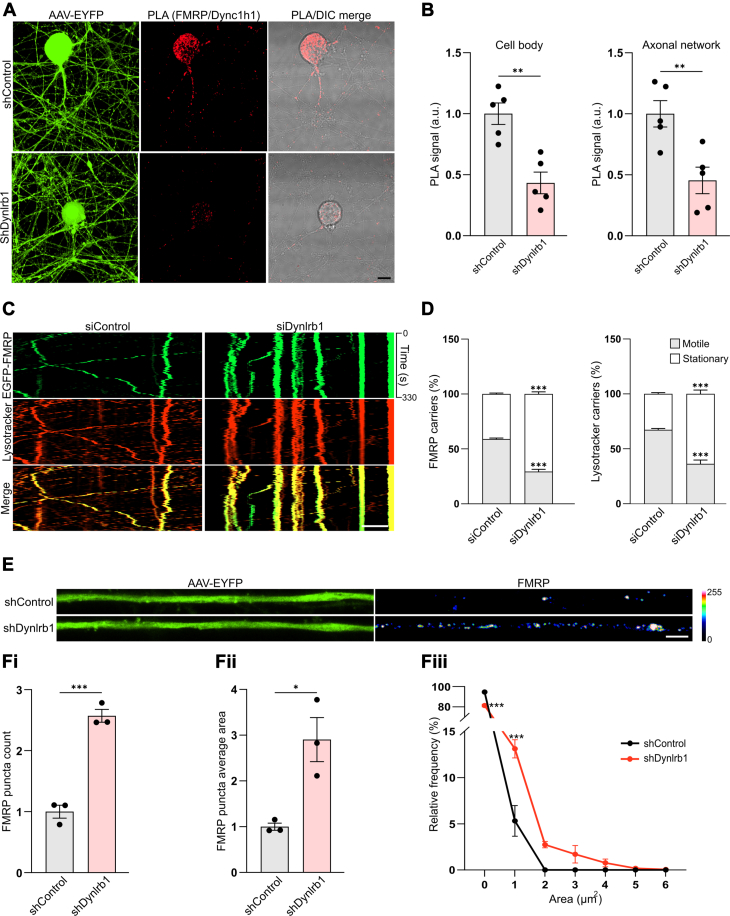
**Genetic depletion of Dynlrb1 negatively impacts FMRP axonal transport and induces axonal FMRP aggregates.***A*, representative images of PLA between FMRP and dynein heavy chain (Dync1h1) in DRG neurons transduced with shControl or shDynlrb1 constructs. Transduced neurons are labeled by EYFP expressed by the viral constructs. The PLA signal is in *red*. Scale bar represents 10 μm. *B*, quantification of the PLA experiment in (*A*). PLA signal in the cell bodies and axons was quantified separately. Mean ± SEM, ∗∗*p* < 0.01, n = 5, unpaired *t* test. *C*, representative kymographs of siControl and siDynlrb1 DRG neurons transfected with EGFP-FMRP construct (in *green*). LysoTracker was added to track lysosomal transport (in *red*). The merge between the two channels is in *yellow*. Scale bar represents 5 μm. *D*, percentage of moving *versus* stationary carriers in the experiment described in (*C*) for both FMRP and LysoTracker-positive axonal carriers. Mean ± SEM, ∗∗∗*p* < 0.001, n = 3, two-way ANOVA, followed by Sidak's multiple comparisons test. *E*, representative images of axons of cultured DRG neurons transduced with shControl or shDynlrb1 constructs. Transduced neurons are labeled by EYFP expressed by the viral constructs. FMRP axonal accumulation is revealed by staining with an anti-FMRP antibody (rainbow palette to highlight difference in intensity). Scale bar represents 5 μm. *F*, quantification of the number (*F*i), area (*F*ii), and relative frequency (*F*iii) of FMRP-positive puncta in the experiment described in (*E*). Mean ± SEM, ∗*p* < 0.05, ∗∗∗*p* < 0.001, n = 3, unpaired *t* test. DRG, dorsal root ganglia; EYFP, enhanced YFP; FMRP, fragile X messenger ribonucleoprotein 1; PLA, proximity ligation assay.

To test the role of Dynlrb1 in FMRP trafficking, we designed an shRNA against the coding sequence of Dynlrb1. Quantitative RT–PCR from cultured DRG neurons transduced with AAV shDynlrb1 showed 60% reduction in Dynlrb1 mRNA levels compared with a non-targeting shControl (supplemental Fig. S4*A*). Given the critical role of Dynlrb1 in sensory neuron survival, we monitored our cultures for signs of toxicity. Six days after transduction with AAV shDynlrb1, only minimal toxicity (8.5% reduction in the number of viable cells) was observed (supplemental Fig. S4, *B* and *C*). We then proceeded to test whether shRNA-mediated knockdown of Dynlrb1 affects FMRP pairing with the dynein complex. PLA analysis revealed that the interaction of FMRP with dynein heavy chain (Dync1h1) (Fig. 4, *A* and *B*) is greatly reduced upon Dynlrb1 depletion suggesting that FMRP active transport could be dependent on Dynlrb1. Accordingly, we tested whether Dynlrb1 depletion would stall FMRP trafficking, inducing its axonal accumulation. Since our viral shRNA constructs express EYFP, we used an siRNA pool to test whether Dynlrb1 is required for EGFP-FMRP axonal motility. Knockdown efficiency was confirmed by quantitative RT–PCR (supplemental Fig. S4*D*). As expected, depletion of Dynlrb1 *via* siRNA significantly increased the stationary pool of lysosomes at the expense of the mobile pool as previously described (19) (Fig. 4, *C* and *D* and Movie S1). Comparably, Dynlrb1 depletion impaired FMRP retrograde transport by increasing the number of stationary particles (Fig 4, *C* and *D* and supplemental Fig. S5*A*; Movie S1) and further reduced the speed of both LysoTracker- (supplemental Fig. S5, *B* and *C*) and FMRP-positive residual retrogradely moving carriers (supplemental Fig. S5, *D* and *E*). Interestingly, EGFP-FMRP stationary particles colocalized with stationary lysosomes (Fig. 4*C*), thus confirming that the long-range transport of lysosomes and FMRP is closely linked and suggesting association between the two.

In line with impaired FMRP trafficking, we observed a significant increase in the number of endogenous FMRP puncta in axons of shDynlrb1-transduced neurons (Fig. 4, *E* and *F*i). Dynlrb1 knockdown also perturbed FMRP axonal puncta size, inducing a statistically significant shift to larger puncta (Fig. 4*F*ii and iii). Super-resolution imaging of the enlarged FMRP puncta revealed them to be clusters of individual FMRP granules (supplemental Fig. S6, *A*–*D*). Further analysis of FMRP puncta from super-resolution images confirmed a statistically significant increase in the number of puncta and a shift to a larger size (0.25–0.75 μm^2^) (supplemental Fig. S6*E*) in Dynlrb1 knockdown neurons. The abnormally enlarged axonal pool of FMRP in Dynlrb1-depleted neurons could result from an impairment in trafficking and/or degradation.

To investigate whether the axonal transport deficit found in Dynlrb1-depleted neurons is specific to lysosomes and FMRP granules, we monitored mitochondrial dynamics. Mitochondria are shuttled bidirectionally by molecular motors, and alterations of their axonal transport have been linked to neurodegenerative diseases (69, 70). DRG neurons transfected with siControl or siDynlrb1 were stained with MitoTracker Deep Red to visualize active mitochondria. Time-lapse imaging revealed a reduction in the motile pool of mitochondria from 32 to 23% upon Dynlrb1 depletion (supplemental Fig. S7, *A* and *B*) with slower movements occurring more frequently despite not impacting the overall median speed (supplemental Fig. S7, *C*–*F*). Interestingly, mutant FMRP was previously reported to negatively impact mitochondrial transport in *Drosophila* axonal network without disrupting their speed of transport (71). Previously, we have also reported that Dynlrb1 genetic depletion negatively affects the percentage of retrogradely motile signaling endosomes but not their speed (19). Remarkably, while our data highlight how Dynlrb1 silencing affects a broad range of cargos, lysosomes are affected to a much greater extent (Fig 4, *C* and *D* and supplemental Fig. S5 *versus* supplemental Fig. S7).

### Dynlrb1 is a Regulator of FMRP Protein Levels and Function

**Fig. 5:**
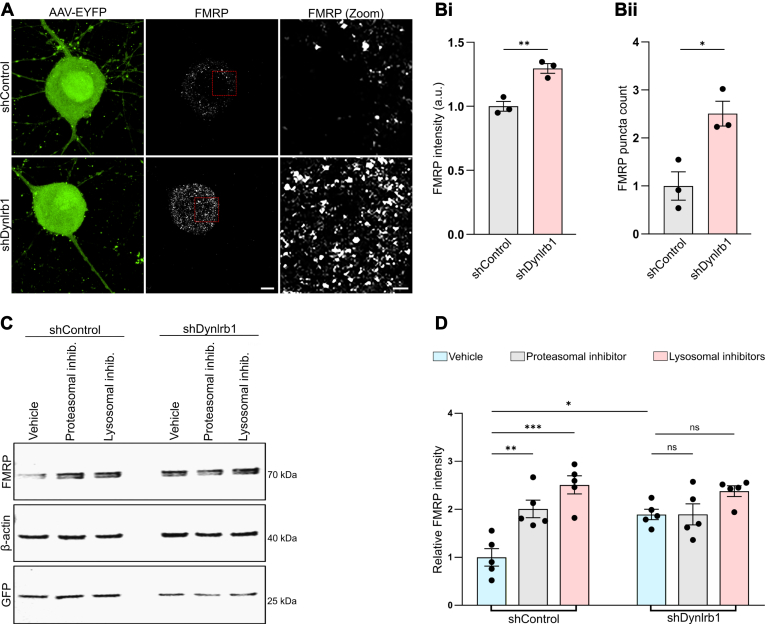
**Genetic depletion of Dynlrb1 impairs FMRP degradation.***A*, representative images of cell bodies of cultured DRG neurons transduced with shControl or shDynlrb1 constructs. Transduced neurons are labeled by EYFP expressed by the viral constructs (in *green*). FMRP granules are visualized by staining with an anti-FMRP antibody (*grayscale)*. Scale bars represent 5 μm and 1 μm, respectively. *B*, quantification of FMRP intensity (*B*i) and the number of FMRP-positive granules (*B*ii) in the experiment described in (*A*). Mean ± SEM, ∗*p* < 0.05, ∗∗*p* < 0.01, n = 3, unpaired *t* test. *C*, Western blot analysis of FMRP protein levels in shControl or shDynlrb1 DRG neurons after incubation with proteasomal inhibitor (MG132), lysosomal inhibitors (leupeptin, pepstatin A, and E-64d) or vehicles for 6 h. β-actin immunostaining was used to normalize FMRP levels. Viral transduction efficiency was visualized by an anti-GFP antibody. *D*, quantification of the experiment described in (*C*). Mean ± SEM, n = 5, ∗*p* < 0.05, ∗∗*p* < 0.01, ∗∗∗*p* < 0.001, ns not significant, one-way ANOVA followed by Tukey’s HSD post hoc correction for multiple comparisons. DRG, dorsal root ganglia; EYFP, enhanced YFP; FMRP, fragile X messenger ribonucleoprotein 1; HSD, honestly significant difference test.

As previously discussed, interaction between BicD and FMRP has been documented in *Drosophila*, and the absence of BicD has been reported to cause a significant reduction of FMRP protein levels in *Drosophila*’s larval brain (36). However, mutations of the dynein motor, which might potentially alter FMRP trafficking, did not affect its protein levels in *Drosophila* (36, 72), suggesting that the non-catalytic subunits of the dynein complex might play a direct role in regulating FMRP protein levels. Thus, we tested whether Dynlrb1 knockdown affects FMRP protein level in DRG neurons. Indeed, Dynlrb1 genetic depletion increased the overall FMRP protein levels (Fig. 5, *A* and *B*i) and further promoted the formation of FMRP granules in DRG neuron soma (Fig. 5, *A* and *B*ii). Since LLPS is facilitated by the local concentration of RBPs (37), the observed increase in FMRP levels could have promoted or enhanced its condensation into granules upon Dynlrb1 silencing. FMRP has been reported to promote the formation of stress granules (37), and aberrant stress granule formation has been linked to neurodegeneration (73). Thus, we examined whether the observed increase in FMRP levels could trigger the assembly and/or accumulation of stress granule in DRG neurons depleted of Dynlrb1. Stress granules were visualized by immunofluorescence staining of G3bp1, one of the most abundant RBPs that is crucial for stress granule formation (74, 75). Indeed, Dynlrb1 knockdown induced a significant increase in G3bp1 level (supplemental Fig. S8, *A* and *B*). Moreover, quantification of G3bp1 intra-axonal signal revealed a significant increase in the number of G3bp1 granules without significantly impacting their size (supplemental Fig. S8*C*).

We then tested whether the loss of Dynlrb1 impairs FMRP degradation by Western blot analysis. Neuronal cultures transduced with shDynlrb1 or shControl were incubated for 6 h with the proteasome inhibitor (MG132) or a cocktail of lysosomal inhibitors (leupeptin, pepstatin A, and E-64d) before protein extraction. Interestingly, both proteasomal and lysosomal inhibition led to a 2- to 2.5-fold increase in the levels of FMRP in shControl neurons (Fig. 5, *C* and *D*). In contrast, MG132 failed to elicit any significant increase of FMRP levels in shDynlrb1 neurons, whereas lysosomal inhibitors induced a minimal but non-significant increase in FMRP protein levels (Fig. 5, *C* and *D*). These data suggest that both the proteasomal and lysosomal pathways work hand in hand in adult sensory neurons to tightly control FMRP level and that FMRP degradation capacity is saturated in DRG neurons depleted of Dynlrb1.

**Fig. 6:**
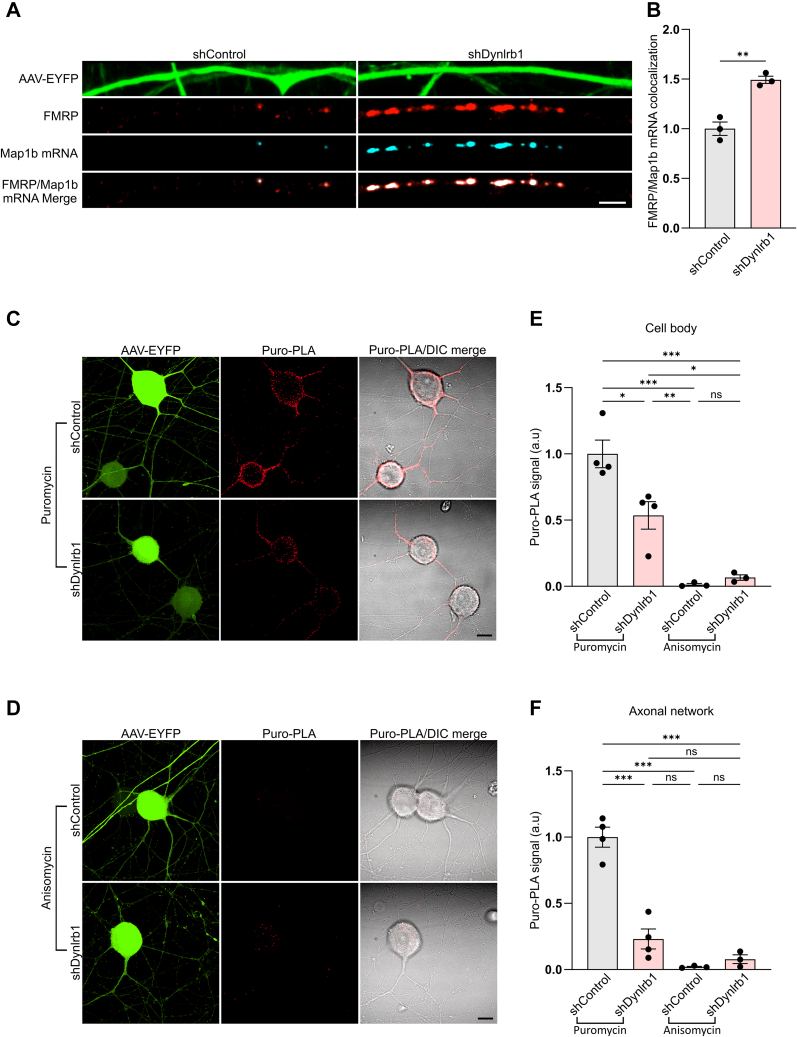
**Dynlrb1 silencing impairs Map1b translation.***A*, representative images of integrated *in situ* hybridization (ISH) for Map1b mRNA and FMRP immunostaining in DRG neurons transduced with shControl or shDynlrb1. Transduced neurons are labeled by EYFP expressed by the viral constructs (in *green*). ISH signal is in *turquoise*. Scale bar represents 5 μm. *B*, quantification of the axonal colocalization between FMRP and Map1b mRNA in the experiment described in (*A*). Mean ± SEM, n = 3, ∗∗*p* < 0.01, unpaired *t* test. *C*, representative images of newly synthesized Map1b in puromycin-treated shControl or shDynlrb1 DRG neurons detected by PLA between anti-puromycin and anti-Map1b antibodies. Neurons were incubated with puromycin for 10 min to label newly translated proteins. Transduced neurons are labeled by EYFP expressed by the viral constructs (in *green*). PLA signal is in *red*. Scale bar represents 10 μm. *D*, representative images of newly synthesized Map1b in anisomycin-treated shControl or shDynlrb1 DRG neurons. Neurons were incubated with 40 μM anisomycin for 30 min prior to incubation with puromycin for 10 min as a negative control for the PLA reaction in (*C*). Transduced neurons are labeled by EYFP expressed by the viral constructs (in *green*). PLA signal is in *red* and detected by PLA between anti-puromycin and anti-Map1b antibodies. Scale bar represents 10 μm. *E* and *F*, quantification of the puro-PLA signal for the experiment described in *C* and *D*, respectively. Mean ± SEM, n ≥ 3, ∗*p* < 0.05, ∗∗*p* < 0.01, ∗∗∗*p* < 0.001, ns not significant, one-way ANOVA followed by Tukey’s HSD post hoc correction for multiple comparisons. DRG, dorsal root ganglia; EYFP, enhanced YFP; FMRP, fragile X messenger ribonucleoprotein 1; HSD, honestly significant difference test; Map1b, microtubule-associated protein 1b; PLA, proximity ligation assay.

We next investigated whether the impaired FMRP degradation, and the consequent dysregulation of its granule formation, could affect the translation of its mRNA targets. We first assessed the localization of the Map1b mRNA, a well-known target of FMRP (76, 77), with FMRP granules using RNA *in situ* hybridization (ISH) combined with immunofluorescence staining. Map1b mRNA exhibited a punctated staining pattern that colocalized with FMRP granules (Fig. 6*A*). The specificity of the Map1b signal was confirmed by the absence of ISH signal upon hybridization of a negative control probe (supplemental Fig. S9*A*). Interestingly, Map1b mRNA sequestration in FMRP granules was increased after Dynlrb1 depletion compared with shControl neurons (Fig. 6, *A* and *B*). Subsequently, we examined the effect of Dynlrb1 depletion on Map1b translation. Direct visualization of newly synthesized Map1b by combining puromycin labeling and PLA revealed a significant reduction in Map1b translation in Dynlrb1 knockdown neurons (Fig. 6, *C*–*F*). The specificity of the PLA signal was determined by pretreatment with 40 μM anisomycin, a protein synthesis inhibitor, as a negative control (Fig. 6, *D* and *F*). Collectively, these results are in accordance with previous literature correlating FMRP condensation with translation inhibition (78, 79), and could, at least in part, explain the impaired axonal outgrowth described in Dynlrb1 heterozygous mouse model (19).

## Discussion

Alterations of the function of dynein (3, 4, 5, 6, 7, 8) as well as the dynactin complex (80, 81, 82, 83, 84) have been linked to motor neuron degeneration and ALS. The dynein heavy chain mutant mouse models *Dync1h1*^*Loa*^ and *Dync1h1*^*Cra1*^ display motor and sensory deficits (85, 86). Likewise, our previous findings indicate that genetic depletion of Dynlrb1 negatively impacts neuronal viability (19). Two roadblock isoforms are expressed in mammalian cells: Dynlrb1 and Dynlrb2. Similar to other subunits (87, 88), isoform diversity could define distinct dynein populations with unique functions as recently reported (87). The broad spectrum of trafficking deficits observed upon Dynlrb1 depletion might indicate a role for Dynlrb1 in the association of endolysosomes and mitochondria with the dynein motor. Alternatively, Dynlrb1 depletion might cause a functional perturbation of a subpopulation of dynein motors. Further investigations and motility reconstitution assay from purified components would be critical to answer this question (10). Previous interactome studies for Dynlrb1 yielded only few hits (17, 89, 90, 91). Nonetheless, the observed neuronal loss upon Dynlrb1 depletion cannot be fully explained by these interactions. Thus, we used a proximity-based proteomics approach to characterize Dynlrb1 interactome and identified the translational repressor FMRP as a putative Dynlrb1’s interacting partner in sensory neurons. FMRP plays a critical role in the spinal sensory system, and sensory deficits have been reported in FXS and FXTAS patients (92, 93, 94). While this work focuses on the characterization of Dynlrb1–FMRP interaction, our MS analysis provides a novel resource for Dynlrb1 neuronal interactome that will be useful in the investigation of dynein-mediated axonal survival signaling (supplemental Tables S1 and S2).

We show how the long-range transport of lysosomes and FMRP granules is closely linked (Fig. 3, *E*–*H*) and dependent on Dynlrb1 (Fig. 4, *C* and *D*). Increasing evidence show how mRNA, miRNA, protein complexes, and RNA granules hitchhike onto membranous organelles for long-range transport (95) and that late endosomes can act as platform for mRNA translation (96). Recently, Lippincott-Schwartz and Ward groups reported that G3bp1 granules hitchhike onto Lamp1-positive organelles utilizing Anxa11 as a tethering adaptor (62). Of note, Anxa11 mutations impacting its propensity to phase separate and its association with lysosomal vesicles have been implicated in familial ALS pathogenesis (68). Our data suggest that FMRP granules utilize the same molecular adaptor to dock on Lamp1-positive organelles (supplemental Fig. S3, *C*–*J*). Endolysosomes represent an ideal platform for the tethering of FMRP granules given the speed and extent of their transport. The pairing of FMRP granules to lysosomes could provide an opportunity for internalization and degradation by microautophagy (62, 97). Indeed, overexpressed FMRP was found inside lysosomal compartments *via* electron microscopy (98), and our own data suggest a block of lysosomal degradation upon Dynlrb1 depletion (Fig. 5, *C* and *D*). Interestingly, our proteomics data identified both Vps29 and Vta1, proteins involved in endosomal and multivesicular body sorting and lysosomal acidification (99, 100, 101), as Dynlrb1’s interactors (supplemental Fig. S2 and supplemental Tables S1 and S2). Furthermore, since FMRP-mediated translation is dependent on synaptic activity, retrograde transport of a pool of RNA granules could support a model in which mRNAs patrol different synaptic sites until being recruited by an active site rather than being permanently anchored at a specific synapse (78, 102). Indeed, FMRP has been shown to be a key regulator of the translation of critical proteins for synaptic signaling and plasticity (29, 34, 35, 103, 104). Retrograde trafficking has also been previously described for G3bp1 and La granules (62, 105). Retrograde transport granules were also proposed to support recycling of mRNAs back to the soma to regulate the copy number of specific axonal mRNA, thereby preserving mRNA:miRNA stoichiometry (106).

Alteration of RNA processing as a result of accumulations of RNP aggregates has been identified as a unifying mechanism underlying the pathogenesis of several neurodegenerative diseases, regardless of the underlying genetic cause (107, 108). We report that Dynlrb1 silencing in sensory neurons promotes aberrant FMRP accumulations that further triggers the formation of stress granule inclusions (supplemental Fig. S8). Interestingly, Dynlrb1 was previously shown to reduce mutant huntingtin (mHtt Q74) and α-synuclein (α-syn A53T) aggregation in mouse brain cells (18), two proteins known for their involvement in neurodegenerative diseases.

Taken together, our data support a pathogenesis model whereby Dynlrb1 silencing promotes FMRP granule formation, hence sequestering FMRP target mRNAs and reducing their availability for translation. Dynlrb1 depletion also stalls FMRP granule trafficking, a process intended to support FMRP clearance and steer spatiotemporal mRNA translation. Accordingly, Dynlrb1 depletion can alter the rather finely tuned translation dynamics in sensory neurons, impacting their functionality and ultimately their survival. Our work expands the understanding of the regulation of the axonal mRNA repertoire and points to axonal transport as another element in defining the correct LLPS equilibrium, thus having a significant impact on neuronal homeostasis, disease pathogenesis, as well as injury response. Nevertheless, further work is necessary to understand the nature of the interaction of FMRP granules with lysosomes and dynein and whether they extend to other RNP granules as well.

## Data Availability

The MS raw data, zipped MaxQuant output folder, MaxQuant software, and the result file have been deposited in the ProteomeXchange Consortium *via* the jPOST partner repository (109) with the dataset identifier PXD039735. URL: https://proteomecentral.proteomexchange.org/cgi/GetDataset?ID=PXD039735

## Supplemental Data

This article contains supplemental data.

## Conflict of interest

The authors declare no competing interests.

## Supplemental Data

Supplemental Table S1 Candidates from the mass spectrometry analysis of Dynlrb1 interacting partners. For each candidate intensity values of the identified peptides are reported for the conditions analyzed. Hits were ranked by intensity MnT-Dynlrb1/ intensity MnT-control.

Supplemental Table S2 Functional analysis of Dynlrb1 putative interactors. GO Terms for biological pathways and molecular functions were obtained using the DAVID bioinformatic database (https://david.ncifcrf.gov/home.jsp) are listed for each candidate.

Movie S1 Time-lapse imaging of EGFP-FMRP (in green) and lysotracker-positive carriers (in red) in DRG neurons. Untreated, siControl, siDynlrb1 neurons are provided respectively. The merge between the two channels is in yellow. Hundred and twenty frames (frame duration of 2.53 s) were consecutively acquired for every time series.
