# Supplementary material for: FMRP Long-Range Transport and Degradation Are Mediated by Dynlrb1 in Sensory Neurons
Source: Mol Cell Proteomics. 2023 Sep 21;22(11):100653. doi: 10.1016/j.mcpro.2023.100653 (PMC10625159; doi:10.1016/j.mcpro.2023.100653)
Supplement: Supplemental Figures S1–S9 [file mmc4.docx]

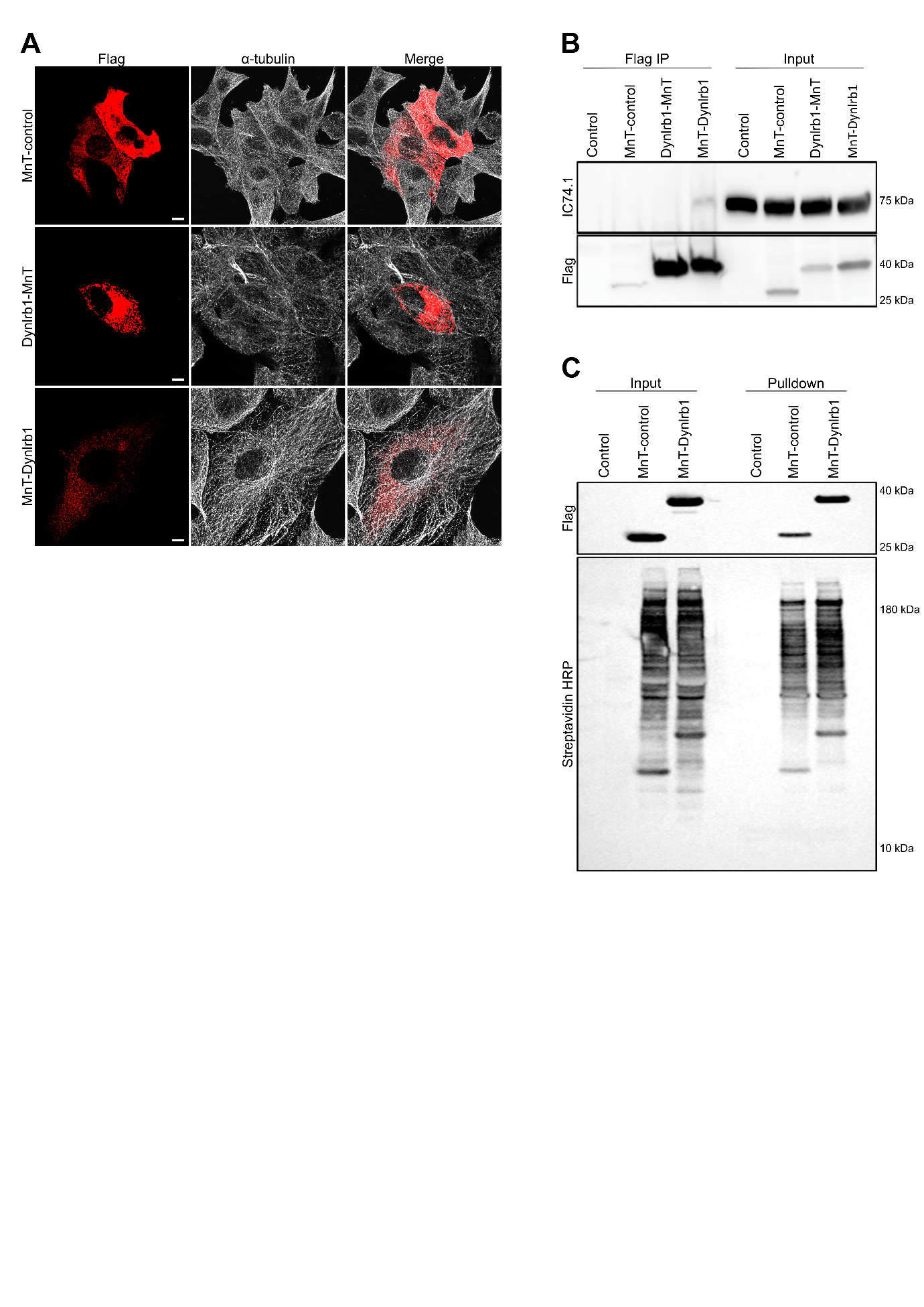


**Supplemental Figure S1**

**Optimization of miniTurbo fusion constructs in 3T3 cells.** *A*, representative images of 3T3 cells transfected with unconjugated miniTurbo (MnT-control) or miniTurbo fused to the C- (Dynlrb1-MnT) or N-terminus (MnT-Dynlrb1) domain of Dynlrb1. The distribution of the flag-tagged proteins in the cytoplasm of transfected cells was revealed by flag staining (in red). Cells were labelled *via* anti-tubulin staining (in gray). Dynlrb1-MnT protein clearly showed aggregation and toxicity. Scale bars represent 10 μm, 5 μm and 5 μm respectively. *B*, immunoprecipitation of flag-tagged proteins from 3T3 cells transfected with MnT-control, Dynlrb1-MnT or MnT-Dynlrb1. Dynein intermediate chain (IC74.1) was used to reveal incorporation in the dynein complex *via* western blot analysis. Non-transfected cells were used as negative control. Only the N-terminal fusion protein (MnT-Dynlrb1) was able to pulldown dynein intermediate chain. *C*, western blot analysis of the streptavidin pulldown of biotinylated proteins in 3T3 cells transfected with MnT-control or MnT-Dynlrb1. The expression is revealed using anti-flag antibody, while the extent of biotinylation is detected using anti-streptavidin HRP. HRP, horseradish peroxidase.


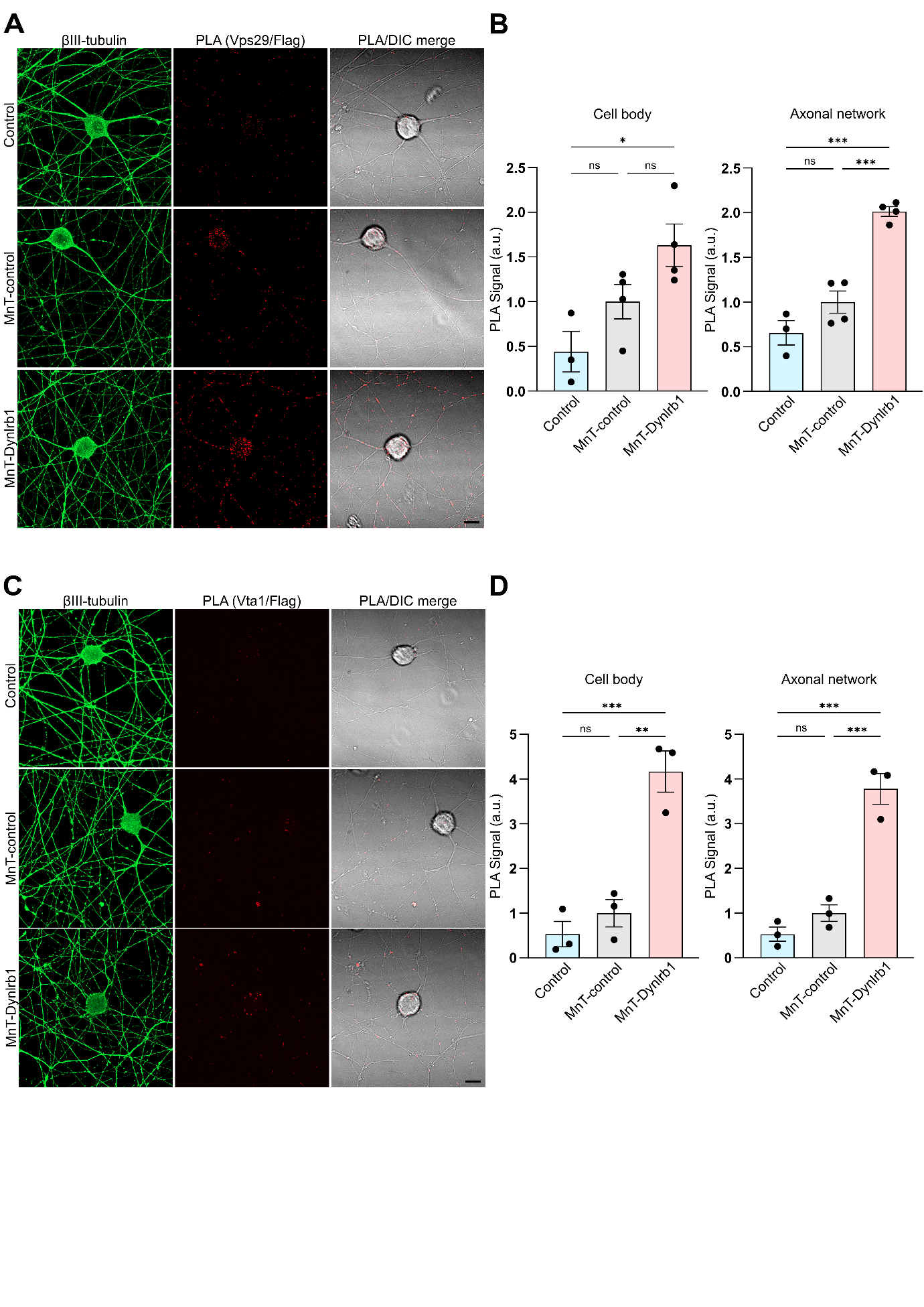


**Supplemental Figure S2**

**Validation of Dynlrb1-Vps29 and Vta1 interaction in DRG neurons.***A*, representative images of PLA between flag-tagged proteins and Vps29 in DRG neurons transduced with miniTurbo or MnT-Dynlrb1 AAV constructs. Non-transduced cells were also included as a control. Neurons are labelled with βIII-tubulin (green). The PLA signal is in red. Scale bar represents 10 μm. *B*, quantification of the PLA experiment in (A). PLA signal in the cell bodies and axons was quantified separately. Mean ± SEM, ∗*p* < 0.05, ∗∗∗*p* < 0.001, ns not significant, n ≥3, one-way ANOVA followed by Tukey’s HSD post hoc correction for multiple comparisons. *C*, representative images of PLA between flag-tagged proteins and Vta1 in DRG neurons transduced with MnT-control or MnT-Dynlrb1. Non-transduced cells were also added as a control. Neurons are labelled with βIII-tubulin (green). The PLA signal is in red. Scale bar represents 10 μm. *D*, quantification of the PLA experiment in (*C*). PLA signal in the cell bodies and axons was quantified separately. Mean ± SEM, ∗∗*p* < 0.01, ∗∗∗*p* < 0.001, ns not significant, n = 3, one-way ANOVA followed by Tukey’s HSD post hoc correction for multiple comparisons. DRG, dorsal root ganglia; HSD, honestly significant difference test; MnT, miniTurbo; PLA, proximity ligation assay; Vps29; vacuolar protein sorting–associated protein 29; Vta1, vacuolar protein sorting–associated protein VTA1 homolog.


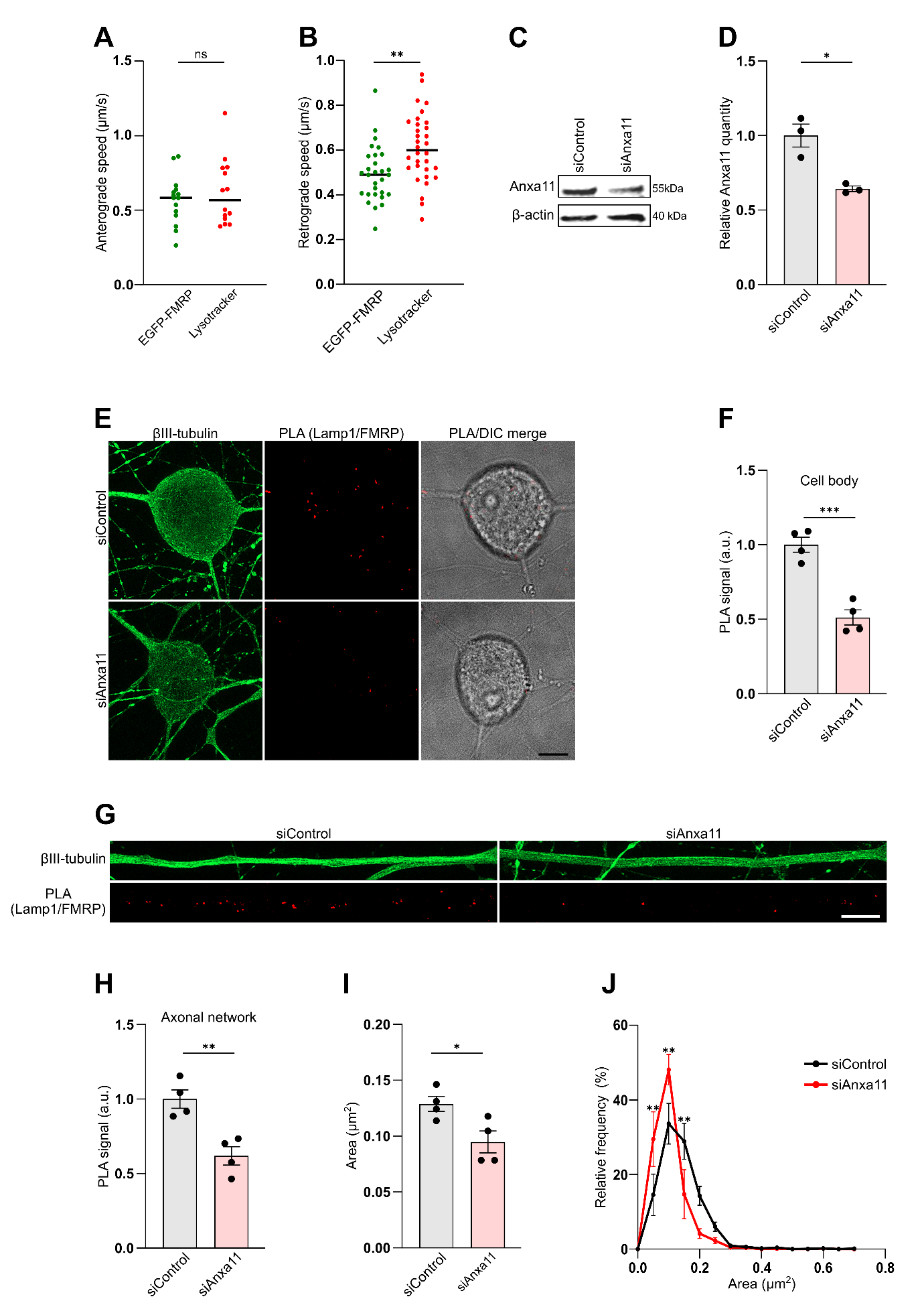


**Supplemental Figure S3**

**Analysis of FMRP trafficking in wild type sensory neurons.***A*, medians of the instantaneous anterograde speeds of moving carriers in the experiment described in ​Figure 3​*E*. Mean ± SEM, ns not significant, n >14 movies per group over three independent biological repeats, unpaired *t* test. *B*, medians of the instantaneous retrograde speeds of moving lysotracker and FMRP carriers in the experiment described in ​Figure 3​*E*. Mean ± SEM, ∗∗*p* < 0.01, n >28 movies per group over three independent biological repeats, unpaired *t* test. *C*, western blot analysis of Anxa11 protein levels in DRG neurons transfected with siControl or siAnxa11. *D*, quantification of the experiment described in (*C*). Mean ± SEM, n = 3, ∗*p* < 0.05, unpaired *t* test. *E*, representative images of PLA between FMRP and Lamp1 in the somatic compartment of siControl and siAnxa11 DRG neurons. Neurons are labelled with βIII-tubulin (green). PLA signal is in red. Scale bar represents 10 μm. *F*, quantification of the PLA experiment in (*E*). Mean ± SEM, ∗∗∗*p* < 0.001, n = 4, unpaired *t* test. *G*, representative images of axonal FMRP/Lamp1 PLA in siControl and siAnxa11 neurons labelled with βIII-tubulin (green). PLA signal is in red. Scale bar represents 10 μm. *H*, quantification of the PLA experiment in (*G*). Mean ± SEM, ∗∗*p* < 0.01, n = 4, unpaired *t* test. *I*, median of the PLA area described in (*E* and *G*). Mean ± SEM, ∗*p* < 0.05, n = 4, unpaired *t* test. *J*, relative frequency distribution for the experiment in (*E* and *G*). ∗∗*p* < 0.01, n = 4, two-way ANOVA followed by Sidak’s multiple comparisons test. Anxa11, annexin A11; DRG, dorsal root ganglia; Lamp1, lysosomal-associated membrane protein 1; PLA, proximity ligation assay.


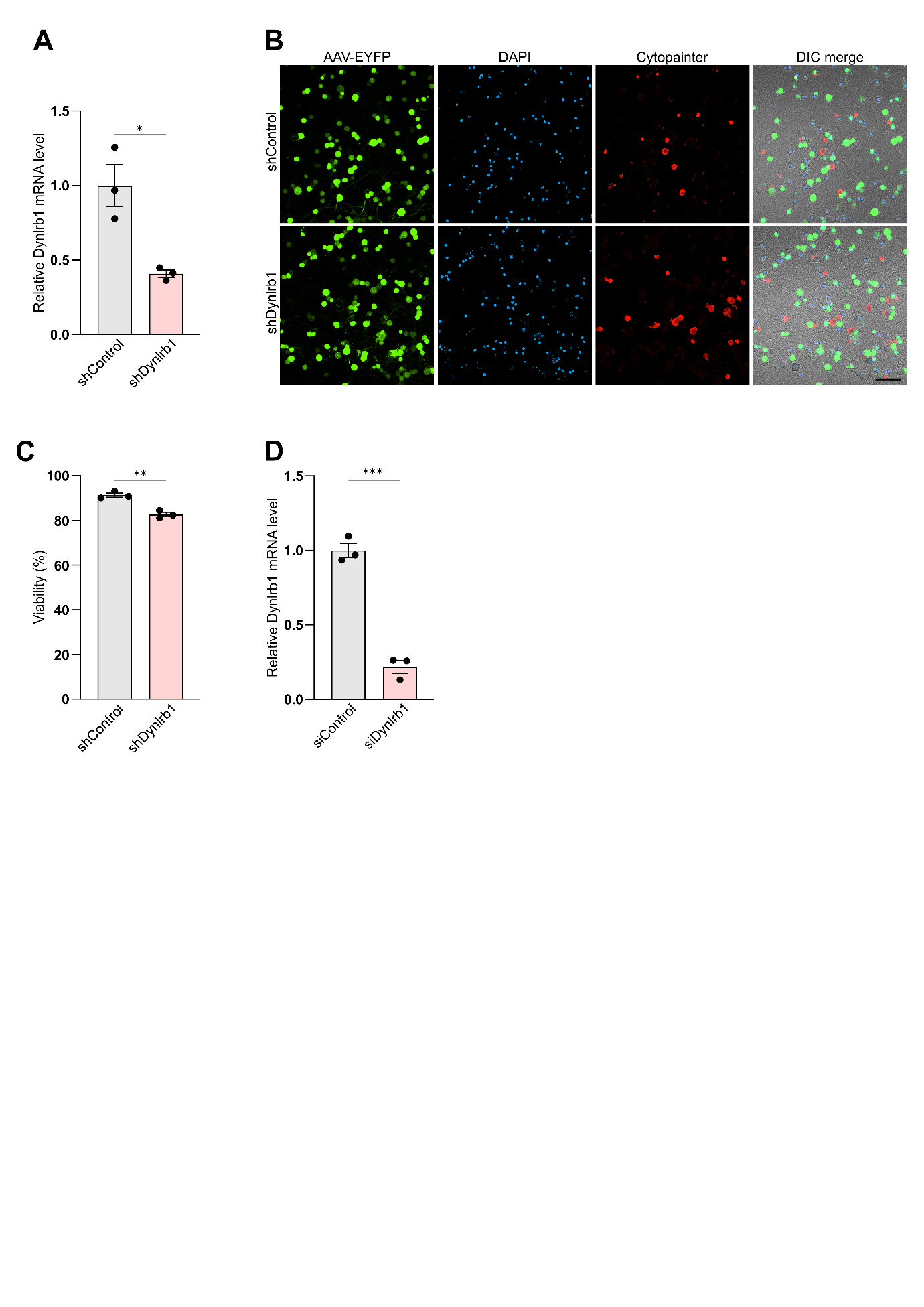


**Supplemental Figure S4**

**Genetic depletion of Dynlrb1 in DRG neurons*.****A*, quantitative RT-PCR analysis on RNA extracted from DRG neurons 8 days post-transduction with shControl or shDynlrb1. Mean ± SEM, ∗*p* < 0.05, n = 3, unpaired *t* test. *B*, representative images of DRG neurons transduced with shControl or shDynlrb1 for 6 days and stained with cytopainter dye for 45 mins. Cells with compromised plasma membranes show high cytopainter fluorescence intensity (in red). Transduced neurons are labelled by EYFP expressed by the viral constructs (in green). Nuclei are visualized by DAPI (in blue). Scale bar represents 100 μm. *C*, quantification of the experiment described in (B). Mean ± SEM, ∗∗*p* < 0.01, n = 3, unpaired *t* test. *D*, quantitative RT-PCR analysis on RNA extracted from DRG neurons 3 days post-transfection with siControl or siDynlrb1. Mean ± SEM, ∗∗∗*p* < 0.001, n = 3, unpaired *t* test. DRG, dorsal root ganglia; EYFP, enhanced YFP.


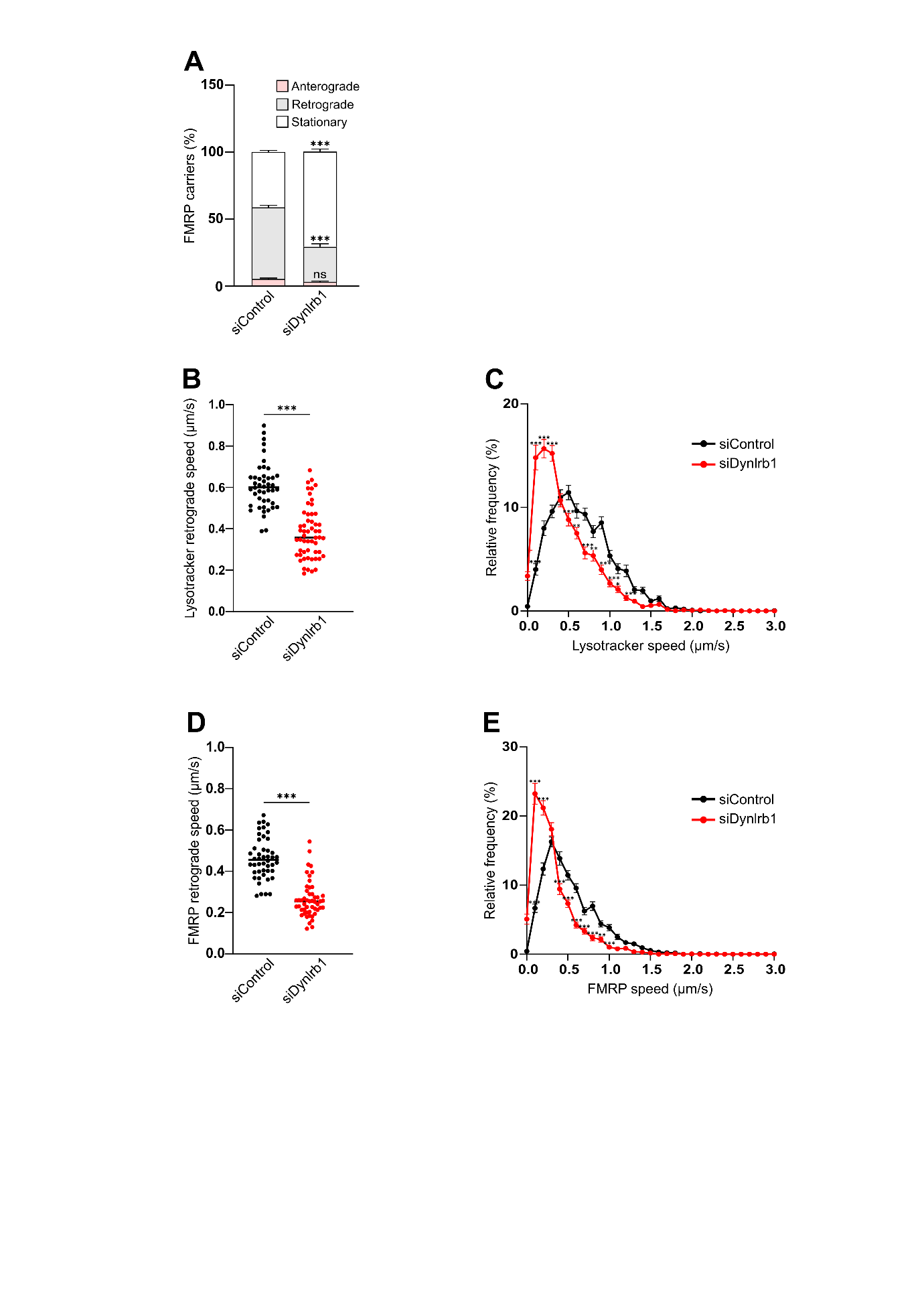


**Supplemental Figure S5**

**Analysis of LysoTracker and FMRP carriers trafficking upon Dynlrb1 depletion.** *A*, percentage of anterograde and retrograde *versus* stationary carriers in the experiment described in ​Figure 4​*C* for FMRP positive axonal carriers. Mean ± SEM, ∗∗∗*p* < 0.001, ns not significant, n = 3, two-way ANOVA, followed by Sidak's multiple comparisons test. *B*, medians of the instantaneous retrograde speeds of LysoTracker-positive moving carriers in siControl and siDynlrb1 DRG neurons transfected with EGFP-FMRP from the experiment described in ​Figure 4​*C*. Mean ± SEM, ∗∗∗*p* < 0.001, n >46 movies per group over three independent biological repeats, unpaired *t* test. *C*, retrograde speed distributions of LysoTracker-positive moving carriers from the experiment described in ​Figure 4​*C*. Mean ± SEM, ∗*p* < 0.05, ∗∗*p* < 0.01, ∗∗∗*p* < 0.001, n >46 movies per group over three independent biological repeats, two-way ANOVA followed by Sidak's multiple comparisons test. *D*, medians of the instantaneous retrograde speeds of FMRP-positive moving carriers in the experiment described in ​Figure 4​*C*. Mean ± SEM, ∗∗∗*p* < 0.001, n >46 movies per group over three independent biological repeats, unpaired *t* test. *E*, retrograde speed distributions of FMRP-positive moving carriers from the experiment described in ​Figure 4​*C*. Mean ± SEM, ∗∗*p* < 0.01, ∗∗∗*p* < 0.001, n >46 movies per group over three independent biological repeats, two-way ANOVA followed by Sidak's multiple comparisons test. DRG, dorsal root ganglia; EGFP, enhanced GFP; FMRP, fragile X messenger ribonucleoprotein 1.


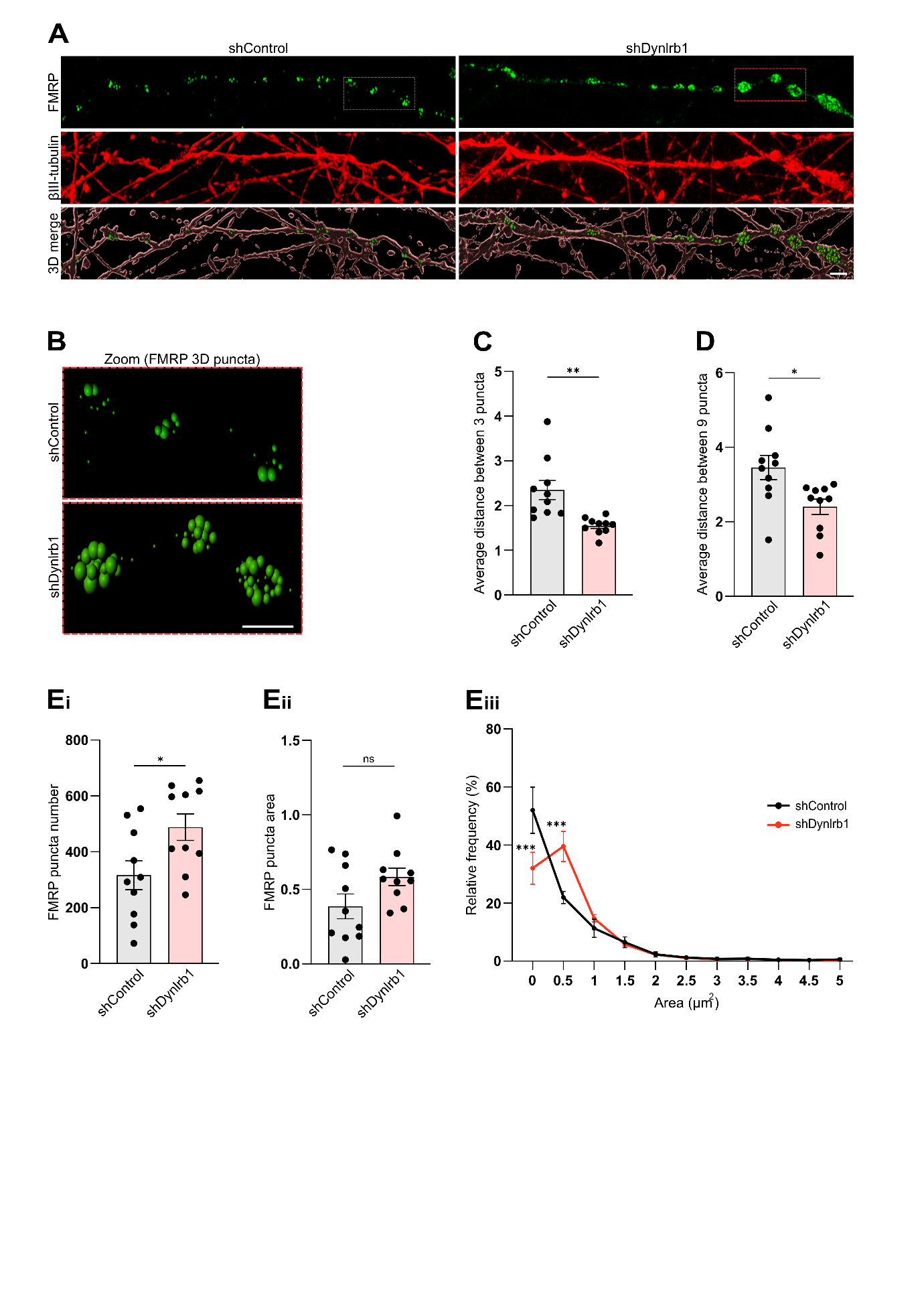


**Supplemental Figure S6**

**Super-resolution imaging of intra-axonal FMRP granules.** *A*, airyscan images of FMRP (in green) and βIII-tubulin (in red), and FMRP 3D spot detection and surface rendering (3D merge) in DRG neurons transduced with shControl or shDynlrb1. Scale bar represents 3 μm. *B*, FMRP puncta detection from the zoom in area shown in panel A (red box). Scale bar represents 3 μm. *C* and *D*, quantification of axonal FMRP clustering with the average distance between 3 spots and 9 spots respectively. *E*, quantification of FMRP puncta number (*E*i), area (*E*ii), and relative frequency (*E*iii). ∗*p* < 0.05, ∗∗∗*p* < 0.001, ns not significant, unpaired *t* test, n = 10 images per group. DRG, dorsal root ganglia; FMRP, fragile X messenger ribonucleoprotein 1.


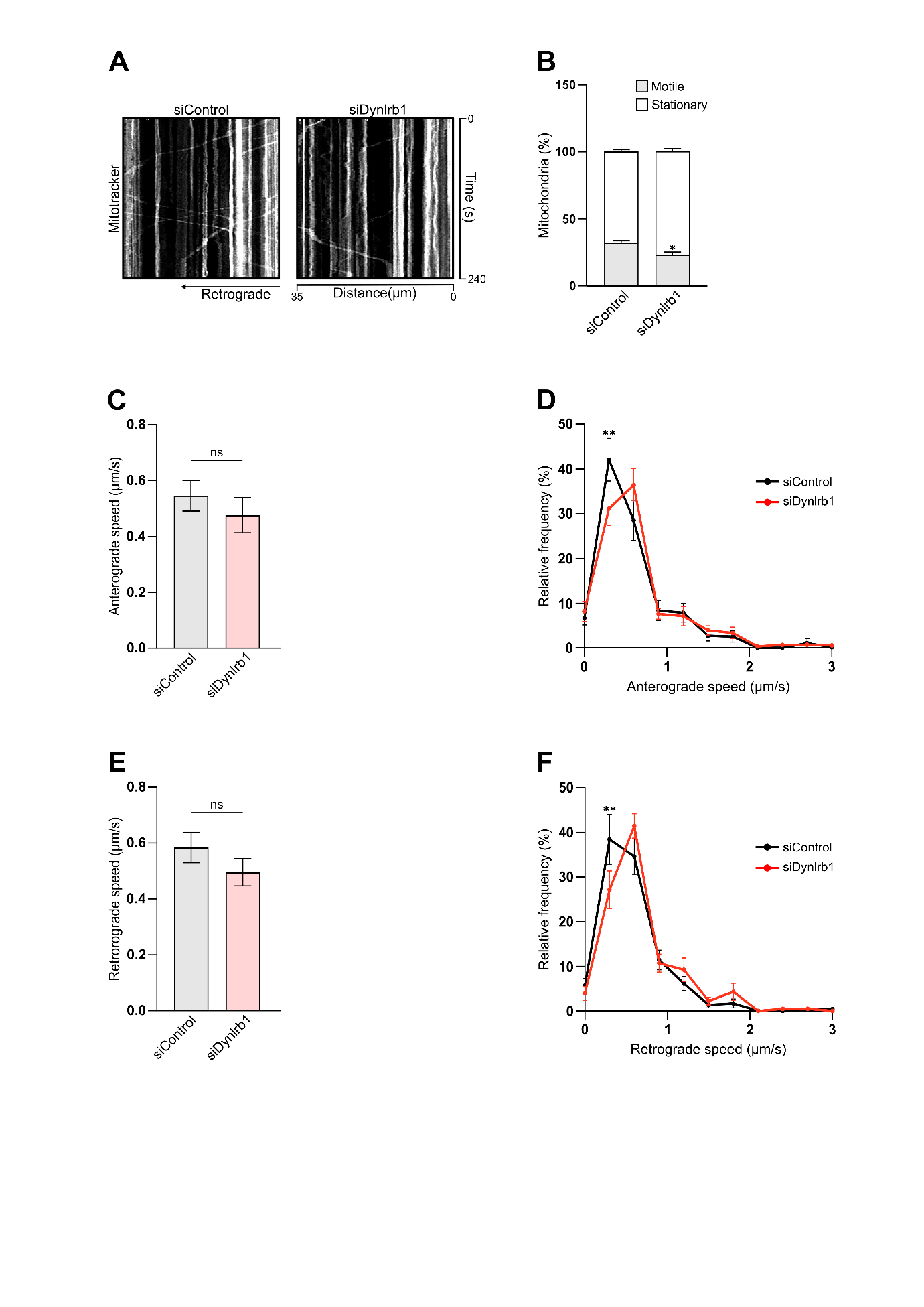


**Supplemental Figure S7**

**Dynlrb1 silencing negatively impacts mitochondrial trafficking.***A*, representative kymographs of siControl and siDynlrb1 DRG neurons labeled with MitoTracker (grayscale). *B*, percentage of moving *versus* stationary carriers in the experiment described in (*A*). Mean ± SEM, ∗*p* < 0.05, n = 3, two-way ANOVA, followed by Sidak's multiple comparisons test. *C*, medians of the anterograde speeds of MitoTracker-positive moving carriers in siControl and siDynlrb1 DRG neurons from the experiment described in (*A*). Mean ± SEM, ns not significant, n > 19 movies per group over three independent biological repeats, unpaired *t* test. *D*, anterograde speed distributions of MitoTracker moving carriers from the experiment described in (A). Mean ± SEM, ∗∗*p* < 0.01, n > 19 movies per group over three independent biological repeats, two-way ANOVA followed by Sidak's multiple comparisons test. *E*, medians of the retrograde speeds of MitoTracker-positive moving carriers in siControl and siDynlrb1 DRG neurons from the experiment described in (*A*). Mean ± SEM, ns not significant, n = 19 movies per group over three independent biological repeats, unpaired *t* test. *F*, retrograde speed distributions of MitoTracker moving carriers from the experiment described in (*A*). Mean ± SEM, ∗∗*p* < 0.01, n = 19 movies per group over three independent biological repeats, two-way ANOVA followed by Sidak’s multiple comparisons test. DRG, dorsal root ganglia.


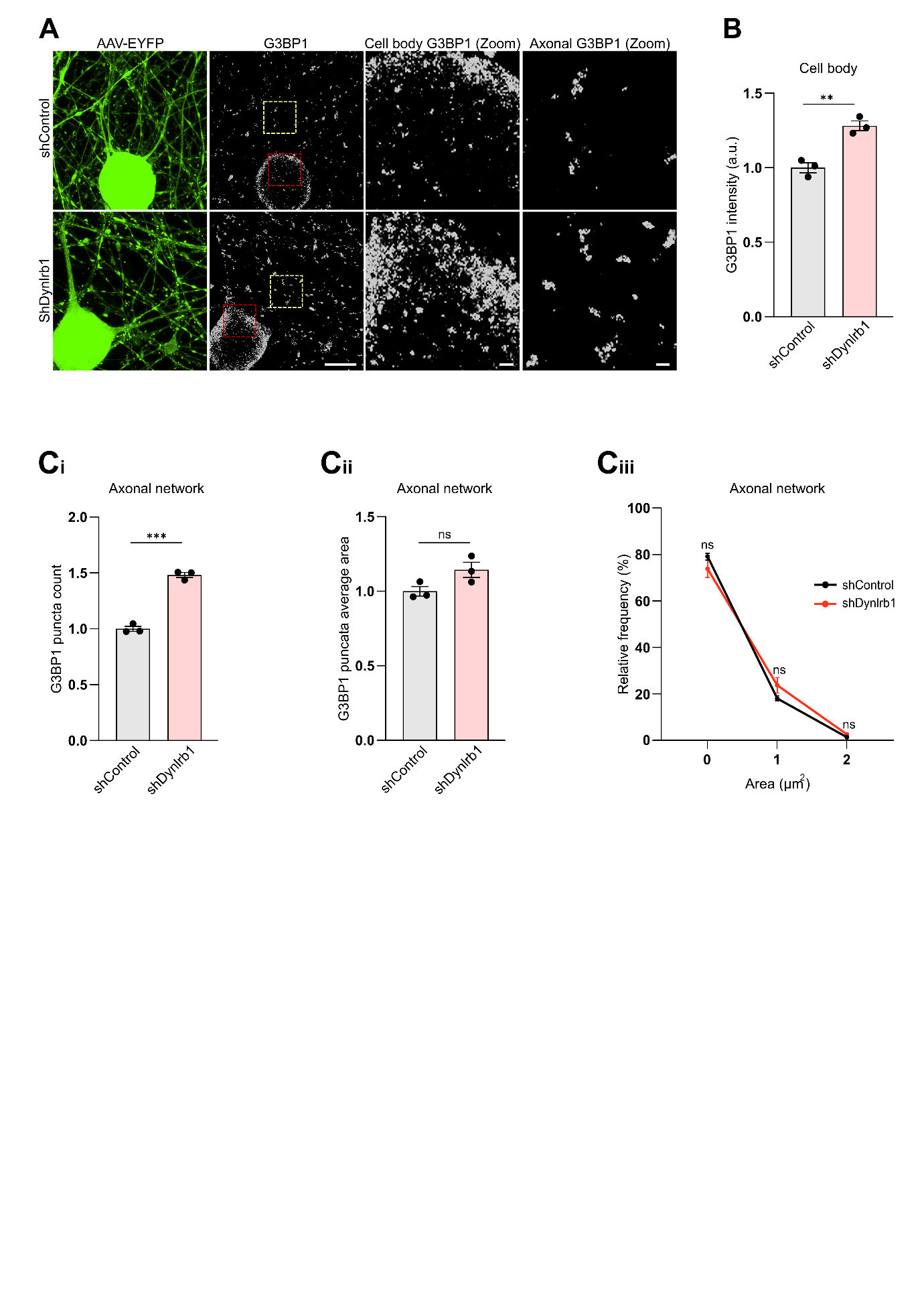


**Supplemental Figure S8**

**Genetic depletion of Dynlrb1 induces stress granule accumulation.***A*, representative images of cultured DRG neurons transduced with shControl or shDynlrb1 constructs. Stress granules are visualized by staining with an anti-G3bp1 antibody (grayscale). Transduced neurons are labelled by EYFP expressed by the viral constructs (in green). Scale bars represent 10 μm, 1 μm and 1 μm respectively. *B*, quantification of G3bp1 intensity in DRG soma for the experiment described in (*A*). Mean ± SEM, ∗∗*p* < 0.01, n = 3, unpaired *t* test. *C*, quantification of the number (*C*i), area (*C*ii) and relative frequency (*C*iii) of axonal G3bp1-positive puncta in the experiment described in (*A*). Mean ± SEM, ∗∗∗*p* < 0.001, ns not significant, n = 3, unpaired *t* test. DRG, dorsal root ganglia; EYFP, enhanced YFP; G3bp1, G3BP stress granule assembly factor 1.


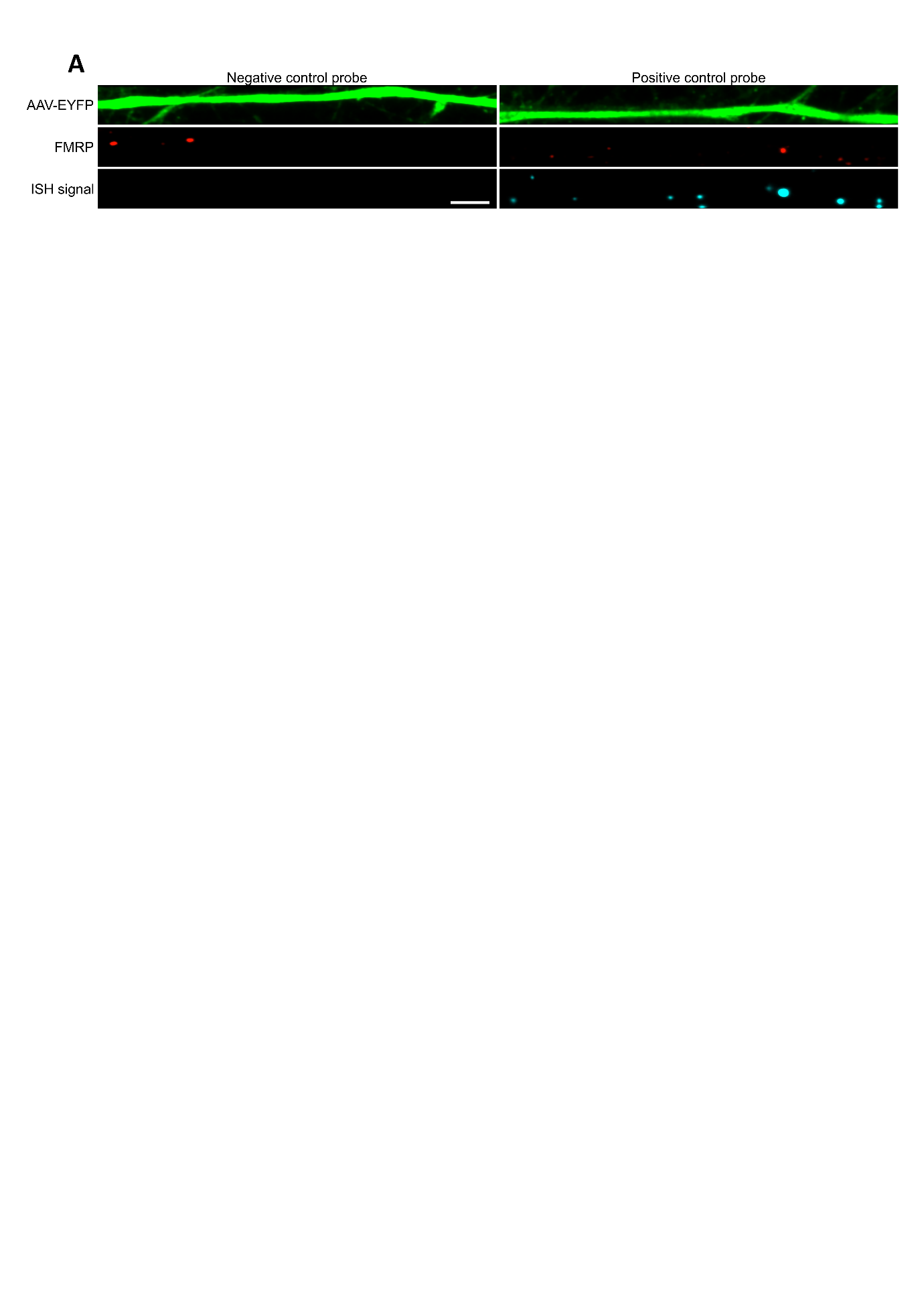


**Supplemental Figure S9**

**RNAscope controls for Map1b mRNA detection in DRG neurons.** (*A*) representative images of *in situ* hybridization signal obtained with negative and positive RNAscope control probes (in Turquoise). Neurons are labelled with EYFP expressed by the viral constructs (in green). FMRP immunostaining signal is in red. Scale bar represents 5 μm. DRG, dorsal root ganglia; EYFP, enhanced YFP; FMRP, fragile X messenger ribonucleoprotein 1; Map1b, microtubule-associated protein 1b.
